# Passenger mutations accurately classify human tumors

**DOI:** 10.1371/journal.pcbi.1006953

**Published:** 2019-04-15

**Authors:** Marina Salvadores, David Mas-Ponte, Fran Supek

**Affiliations:** 1 Institute for Research in Biomedicine (IRB Barcelona), The Barcelona Institute of Science and Technology, Baldiri Reixac, Barcelona, Spain; 2 Institució Catalana de Recerca i Estudis Avançats (ICREA), Barcelona, Spain; University of Maryland Baltimore County, UNITED STATES

## Abstract

Determining the cancer type and molecular subtype has important clinical implications. The primary site is however unknown for some malignancies discovered in the metastatic stage. Moreover liquid biopsies may be used to screen for tumoral DNA, which upon detection needs to be assigned to a site-of-origin. Classifiers based on genomic features are a promising approach to prioritize the tumor anatomical site, type and subtype. We examined the predictive ability of causal (driver) somatic mutations in this task, comparing it against global patterns of non-selected (passenger) mutations, including features based on regional mutation density (RMD). In the task of distinguishing 18 cancer types, the driver mutations–mutated oncogenes or tumor suppressors, pathways and hotspots–classified 36% of the patients to the correct cancer type. In contrast, the features based on passenger mutations did so at 92% accuracy, with similar contribution from the RMD and the trinucleotide mutation spectra. The RMD and the spectra covered distinct sets of patients with predictions. In particular, introducing the RMD features into a combined classification model increased the fraction of diagnosed patients by 50 percentage points (at 20% FDR). Furthermore, RMD was able to discriminate molecular subtypes and/or anatomical site of six major cancers. The advantage of passenger mutations was upheld under high rates of false negative mutation calls and with exome sequencing, even though overall accuracy decreased. We suggest whole genome sequencing is valuable for classifying tumors because it captures global patterns emanating from mutational processes, which are informative of the underlying tumor biology.

## Author summary

Mutations accumulate throughout the lifetime of human somatic cells. While some may affect oncogenes or tumor suppressor genes and cause tumors–the ‘driver’ mutations–most are thought to be of no consequence. The density of such ‘passenger’ mutations across the human chromosomes is very uneven and is correlated with replication time and gene expression in the cell type the tumor had originated from. This property can be used to classify a tumor, assigning it to a tissue of origin and also the molecular subtype. This is useful in cases of those metastatic cancers where the location of the primary tumor is unknown and is also of interest for the upcoming ‘liquid biopsy’ diagnostic approaches, where DNA is directly sequenced from bodily fluids to detect the presence of a cancer. The ability to type and subtype tumors is important to guide more detailed diagnostics and therapy, because the organ and the cell type which generated the tumor determines response to a variety of therapies, including targeted drugs.

## Introduction

The rapid development of genomic techniques has brought considerable advances to the diagnosis and treatment of cancer. Most prominently, genetic variants in cancer genes can serve as markers for targeted therapeutics, in certain cases resulting in an impressive clinical response. Nevertheless, such cases are still not prevalent [1,2], and the cancer tissue-of-origin is also a major factor in deciding on therapeutic approaches [3]. For example, the common V600E mutation in the BRAF oncogene is a marker of the response to the BRAF V600E-targeting drug vemurafenib in melanoma. However, colorectal cancer bearing the same mutation does not respond to the drug [4], illustrating how both the particular oncogenic mutation and the tissue of origin are important for predicting the response to therapy. Consistently, large-scale drug screens on cancer cell lines suggest that drug response is often determined by gene expression patterns that stem from the cancer type [5], independently of the mutated oncogenes. The cancer type classification is being continually refined and extended [6]: driven by large-scale transcriptome, methylome and proteome analyses, new molecular subtypes are being proposed for various cancers [7–9]. Because these subtypes may have important clinical implications such as survival differences, it is advantageous that the cancer type and subtype are accurately determined for each patient. Genome sequencing provides an opportunity for development of approaches to meet this need, yielding cancer classifiers which may be useful to guide diagnostic work and decisions on treatment in certain scenarios. First, in approximately 3% of metastatic cancers the standard diagnostic procedure cannot identify the site of origin [10]. While there exist algorithms to classify such ‘cancers of unknown primary’ (CUP) based on gene expression [11,12] or DNA methylation [13], genome sequencing provides an opportunity to obtain independent predictions. Second, genomic classifiers of cancer type are relevant for liquid biopsies, where cell-free DNA or circulating tumor cells are retrieved from blood and DNA sequencing is performed. Recently, this approach was shown to hold much potential for screening the general population, or persons at risk for cancer [14,15]. After having determined that a tumor may be present, the genomic data from a liquid biopsy may be used to prioritize suspected primary sites, minimizing invasive diagnostic tests and reducing the time to therapy. Third, genomic classifiers may be valuable in the common case where the primary site of the tumor is known, because mutational patterns provide an additional means to determine the molecular subtype of the tumor. Standard subtyping is based on histopathology and immunochemistry tests and on gene expression panels. However, it is increasingly appreciated that integrating diverse omics data types leads to more robust subtyping [6,16], motivating research into how somatic mutation data may complement transcriptome, DNA methylation or proteome data for assigning a clinically relevant subtype to each tumor [17].

Therefore, a class of emerging approaches aims to classify cancers based on somatic mutations observed by comparing genomes of tumor and healthy tissue from the same patient. This genomic data is complex and features useful for classification need to be extracted from it. One important set of features describe the presence of specific driver mutations in a particular tumor. These mutations are positively selected during tumor evolution because they promote growth of the mutated clones, meaning they are causal to carcinogenesis. Cancer driver genes affected by mutations are known to differ between tissues, where for instance the KRAS oncogene is often mutated in pancreatic, lung and colorectal cancer, but rarely in brain, breast and skin cancer. This provides a rationale for use of driver mutations in existing tumor classifiers [15,18].

In addition to a small number of driver mutations, each cancer contains orders of magnitude more passenger mutations, which are not selected during tumor evolution [19]. Because they are not shaped by selection, passenger mutation patterns provide a track record of the mutagenic processes that a tumor has undergone. These differ between tissue of origin, for instance melanoma has a very high proportion of C>T changes resulting from UV radiation [20]. More generally, it has been shown that considering the differential mutability of trinucleotides can provide a ‘mutation signature’ which results from exogenous and endogenous mutagenic exposures and, consistently, varies between cell types [21]. Indeed, trinucleotide and also pentanucleotide mutation frequencies were previously applied as predictive features to classify a tumor to a tissue-of-origin [22,23].

In this work, we examined the predictive utility of these existing features and evaluated a novel set of genomic features, based on the global patterns of passenger mutations: the regional mutation density (RMD). In particular, somatic mutation rates are known to exhibit striking variability at the megabase scale, wherein late-replicating, heterochromatic regions mutate faster due to reduced DNA repair [24–26]. The pattern of RMD, measured in megabase-scale chromosomal domains, is sufficiently variable across tumor types to allow their discrimination. In particular, the domains which change towards earlier replication timing and higher average gene expression in a tissue also mutate less in that tissue [26], and similarly so if their chromatin is more accessible [27]. While these associations do not reveal the causal factors underlying mutability, they nevertheless suggest that global RMD patterns emanating from thousands of passenger mutations are a useful marker for tissue of origin.

Therefore, we have systematically evaluated the ability of the passenger mutation-derived RMD features to classify 18 tumor types and subtypes thereof. Next, we asked if the predictions are complementary to those obtained by established passenger mutation-derived features, the trinucleotide mutation spectra (henceforth: MS96). Finally, we compared both types of passenger features–the RMD and the MS96 –with features describing the occurrence of specific driver mutations in a tumor (oncogenic mutations, OGM). Overall, passenger mutations are substantially more predictive than drivers in the task of classifying cancer type and subtype, and the RMD are an important component of a combined tumor type classifier based on global patterns of passenger mutations.

## Results

### Classification of tumors using global RMD features

**Fig 1:**
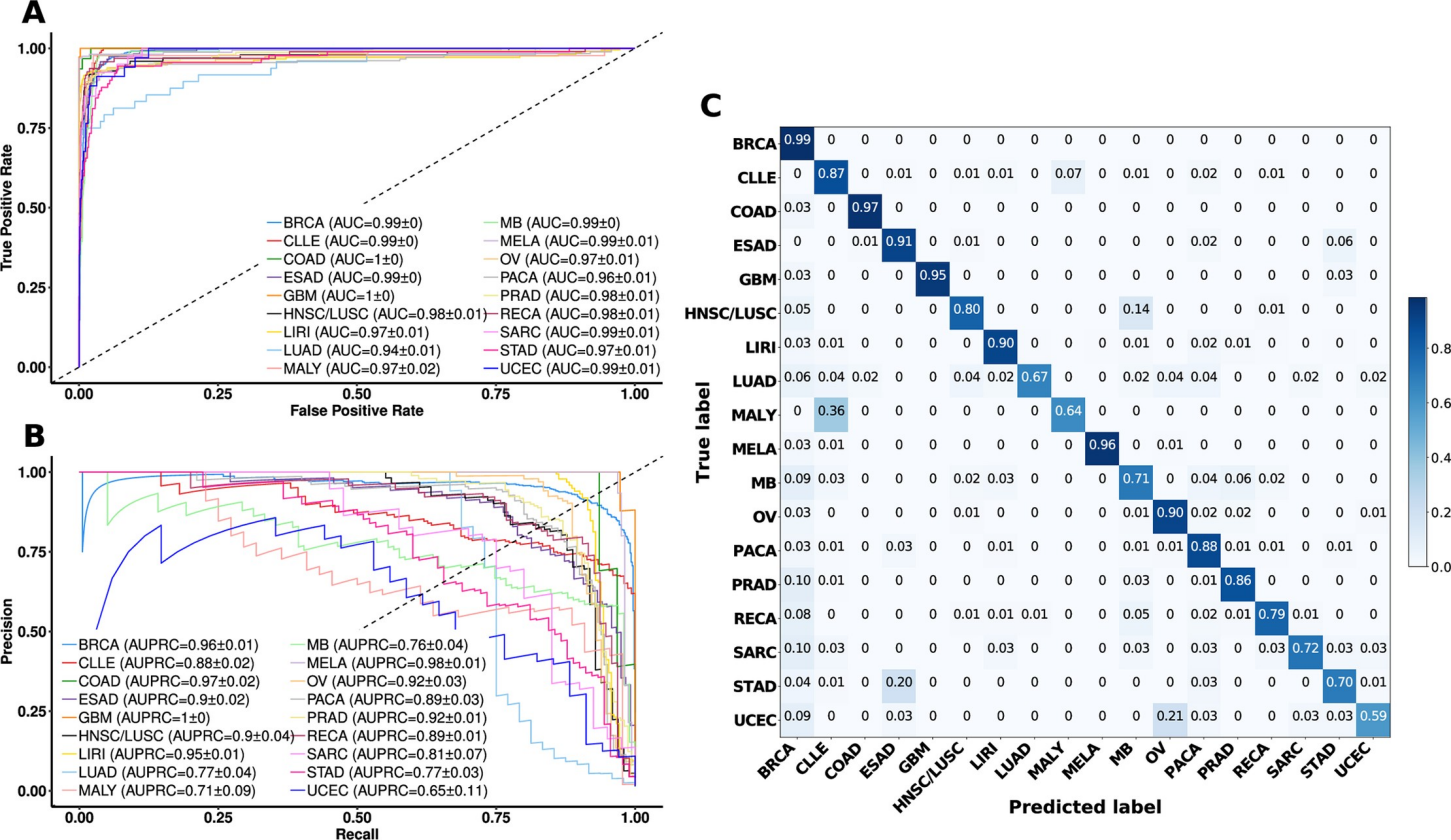
Accuracy of classifiers based on regional mutation density (RMD) in predicting cancer type. (A) Receiver Operating Characteristic (ROC) curve for each cancer type versus the rest. Area under the ROC curve mean and standard error of the mean across crossvalidation folds for each cancer type are reported in the legend. (B) Precision-recall (PR) curve for each cancer type versus the rest. Area under the PR curve mean and standard error of the mean across crossvalidation folds for each cancer type is reported in the legend. (C) Normalized confusion matrix, showing the fraction of misclassified tumors.

We systematically evaluated whether cancer types can be classified using features describing regional mutation density (RMD), which are simply the normalized mutation counts across 2655 megabase-sized chromosomal domains. For this analysis we used a dataset which contains 18,863,479 mutations (SNVs and short indels) from whole-genome sequences (WGS) of 2267 tumor samples from 18 cancer types (S1A Fig and S1 Table). We calculated the RMD features and supplied them to an SVM classifier to generate 18 models that differentiate between each cancer type and the rest (One-vs-Rest scheme). To assess the accuracy of the models, we calculated the Receiver Operating Characteristic (ROC) curve and the area under the ROC curve (AUC) for each cancer type using five-fold crossvalidation (Fig 1A). Classification models for 16 out of 18 cancer types showed a crossvalidation AUC≥0.97, and the two remaining cancer types ≥0.93, indicating RMD are indeed highly informative of cancer type; see S1 Table for a list of cancer type acronyms. While the AUC is a useful measure for comparing the accuracy between data sets with different class sizes (here, ranging from 37 glioblastoma to 560 breast cancer), in case an interpretable accuracy measure for imbalanced datasets is desired, the Area Under the Precision-Recall Curve (AUPRC) is preferable [28,29]. Our models yielded crossvalidation AUPRC scores ranging from 0.65 for UCEC to 1.00 for GBM cancer types (Fig 1B). In addition to AUC and AUPRC measures that integrate over a range of stringency thresholds, we also provide point estimates of precision, recall and F-score for each cancer type (S1B Fig). F-score, the harmonic mean of precision and recall, is very high in certain cancer types (≥0.90 in MELA, GBM, COAD, LIRI and BRCA), while it is lower for MALY (B-cell lymphoma) and UCEC (endometrioid uterine carcinoma), being 0.68 and 0.63, respectively. To investigate the cause for these differences, we examined the confusion matrix (Fig 1C) to ascertain which types of errors are being made. MALY and CLLE (chronic lymphocytic leukemia) were confounded by the models, consistent with both being blood cancers derived from B-cells. Next, STAD and ESAD are also confounded, which is consistent with the observation that the esophageal tumors close to the gastro-esophageal junction have molecular characteristics similar to gastric adenocarcinoma [30]. UCEC is the cancer type confounded with another gynecological cancer, OV (ovarian serous adenocarcinoma), suggesting our classifiers might capture gender-specific features. Overall, many of the apparent errors of the RMD classification models reflect genuine tumor biology and not noise and/or biases which might stem from how the genomic data was obtained or processed.

To further investigate the known sources of bias, we evaluated if the models are influenced by batch effects (sequencing center, age and ancestry) by examining the first five principal components (PCs) of a PC analysis of the RMD features (S2–S4 Figs). The tumor samples tend to cluster by cancer type, but the samples from different sequencing centers, of different age and ancestry are intermixed (S2–S4 Figs) and do not present an obvious pattern. For these groups, we obtained similar accuracies when training on one group and testing on the others, *versus* training and testing in a mixture of the groups (S5 Fig), suggesting the grouping does not confound overall accuracy estimates. Additionally, we validated the models on external data sets, in order to investigate if overfitting is evident in the RMD-based predictive models. For this, we considered all cancer types represented by at least two datasets originating from different sources, distributing them in a secondary training set of 1749 tumor samples and an external validation set of 640 samples, covering 11 cancer types (S2 Table and S6A Fig). We then generated SVM models using the secondary training set and tested it (i) using crossvalidation and (ii) on the external validation dataset (S6B and S6C Fig and S5 Table). Overall, the crossvalidation AUC of 0.99 ± 0.02 matches the external validation set AUC of 0.99 ± 0.04 (median ± IQR across cancer types) implying no detectable overfitting (p-value = 0.22 for AUC difference, Wilcoxon test). Finally, we randomized the labels of the RMD classifiers and obtained AUC scores of approximately 0.5 (S7 Fig), further substantiating that the models do not fit to noise. In summary, the global patterns of passenger mutations captured by the RMD features provide a robust cancer type classifier.

### Classification of cancer molecular subtypes using RMD

**Fig 2:**
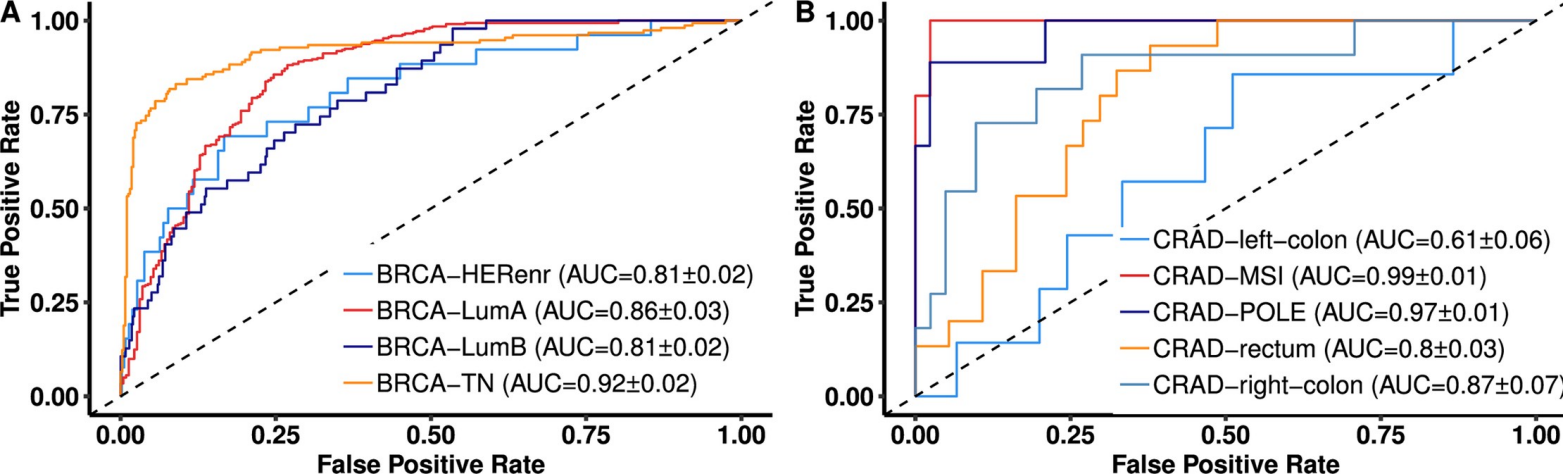
Accuracy of classifiers based on regional mutation density (RMD) in predicting cancer subtypes. Receiver Operating Characteristic (ROC) curves for discriminating each tumor subtype and/or anatomical location, versus the rest within that cancer type, shown for breast cancer (A) and colorectal cancer (B). See S8 Fig for additional cancer types. TPR and FPR calculated in five-fold cross validation.

In addition to classifying cancer type, being able to identify the molecular subtype is important to guide further diagnostics, monitoring or treatment. Therefore, we asked whether the global patterns in RMD could be applied to tumor subtyping. To this end, we trained predictive models to distinguish subtypes for six major cancer types and evaluated them using crossvalidation in the same manner as the between-cancer type classifiers. For breast cancer, the classifier is able to separate the four molecular subtypes with an AUC>0.8 for every subtype and in particular the triple-negative subtype can be recognized more accurately with AUC = 0.92 (Fig 2A). For liver cancer, the model distinguishes biliary and hepatocellular tumors with AUC = 0.77 (S8D Fig). For stomach cancer, apart from its molecular subtypes (diffuse and intestinal) we also included the MSI status as a subtype because it is an indicator of response to cytotoxic chemotherapy such as 5-FU as well as immunotherapy [31]. All three subtypes are classified with AUC≥0.80, particularly the MSI subtype with AUC≈1 (S8F Fig), which is expected given the broad disruption of RMD previously observed in MSI cancers [26]. We note that the MSI subtype can be more directly observed by quantifying microsatellite indels in genomic data without necessity for RMD analysis, but it nonetheless provides a demonstration how RMD may be a useful tool for subtyping of tumors. Similarly to stomach cancer, we divided colorectal cancer by anatomical location (left *versus* right colon) and also based on the presence of MSI and POLE hypermutation, which have therapeutic relevance, yielding AUCs of 0.61 to 0.99 (Fig 2B). The modest AUC of 0.61 of left *versus* right colon suggests it might be possible to use RMD to classify intestinal tumors based on anatomical location, but given the small sample size this is not a significant result (p = 0.14, Mann-Whitney test on AUC>0.5). In case of head-and-neck squamous cell carcinoma and of melanoma, RMD clearly separated the cancer subtypes by anatomical location: oral *versus* non-oral groups (incl. alveolar ridge, larynx, oropharynx and tonsil) at AUC = 0.89 and the three anatomical sites of melanoma at AUC ≥ 0.83 for each (S8E and S8F Fig).

In summary, the global mutational patterns across the genome are systematically different for the subtypes and anatomical locations within the same cancer type in a manner which can be recognized by RMD classifier.

### Predictive power of driver *versus* passenger mutations

As described above, other genomic features have been reported to be able to classify cancer types [18,22,23]. These consist of features that are–similarly to RMD–derived from global patterns of passenger mutations, in particular the relative frequency of mutation types sorted by trinucleotide mutation spectra, herein referred to as MS96 (mutation spectrum with 96 components). In addition, the previously utilized features also include those derived from occurrence of cancer driver mutations (herein referred to as OGM, for oncogenic mutations). We turn to compare the accuracy of models based on novel RMD features with the models based on MS96 and based on OGM in their ability to distinguish cancer type, subtype and anatomical location.

**Fig 3:**
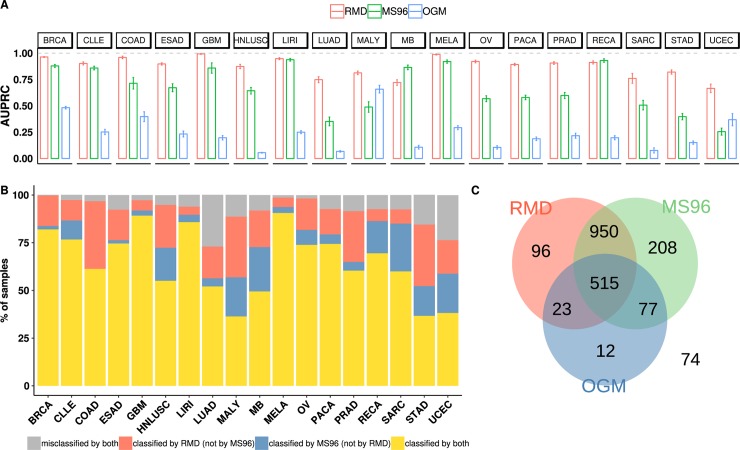
Predictive accuracy and complementarity of driver and passenger mutation features. (A) Mean Area Under the Precision-Recall Curve (AUPRC) for each cancer type, shown for classifiers derived from: regional mutation density (RMD, in red), trinucleotide mutation spectra (MS96, in green) and presence/absence of driver oncogenic mutations (OGM, in blue). Error bars are S.E.M. (B) Proportion of tumor samples that are: correctly classified by both RMD and MS96 (yellow), misclassified by both methods (gray), correctly classified by the MS96 but not by the RMD (blue) and vice versa (red). (C) Overlap between tumor samples correctly recognized by classifiers based on RMD, MS96 or OGM features independently.

First, we evaluated how each set of features performed individually in terms of the crossvalidation AUPRC (Fig 3A). Strikingly, the genomic features derived from global, genome-wide patterns of passenger mutations (RMD and MS96) performed notably better than the presence/absence of known driver mutations (OGM). In particular, passenger RMD yielded an AUPRC of 0.90 ± 0.13 (median ± IQR across 18 cancer types), passenger MS96 yielded 0.66 ± 0.40, while driver OGM only 0.21 ± 0.16, when using a Support Vector Machine classifier.

To ascertain this result is not specific to the SVM classifier, we repeated the above analyses by applying a Random Forest algorithm, which yielded crossvalidation AUPRC of 0.70 for RMD (median across cancer types), 0.77 for MS96 and 0.23 for driver OGM features. This supports the notion that RMD features are informative using various algorithms, but also highlights the utility of the SVM in producing most accurate models possible using this data. Moreover, the RMD features are more numerous than others, and we thus investigated if this affects the results. Upon removing correlated RMD features (see Methods) to reduce their number to 500 –thus matching the OGM–the predictive accuracy of the RMD remained high (median AUPRC = 0.80; S9 Fig), still exceeding MS96 and OGM. We supply the most highly ranking RMD, MS96 and OGM features for every cancer type in S7 Table. The use of feature selection prior to training the SVM classifiers did not improve the accuracy of the models (S10 Fig).

The fact that oncogenic driver mutations (OGM) were, in relative terms, poorly discriminative across cancer types suggests that no combination of oncogenes or tumor suppressor genes is uniquely informative of each individual cancer type, even though many cancer genes are well known to be enriched in certain groups of cancer types. We note that the classification algorithm we use (SVM using a Gaussian kernel) is capable of modeling statistical interactions between features, meaning it can in principle draw on epistatic effects between driver mutation occurrence that are cancer type-specific [32]. We recognize however that many cancer genes are mutated infrequently [33] or may be specific to some subtypes of a cancer type [7,8] and therefore the number of instances in the training dataset might be limiting for recovering complex patterns linking driver mutations and cancer site. We therefore examined the performance of the OGM features on a larger set of 6403 whole-exome sequences from the 15 cancer types matched to the original dataset (1971 WGS in those types). Indeed this did result in an improvement (AUPRC 0.54 ± 0.47 versus 0.24 ± 0.15, respectively, median ± IQR), but the accuracy nevertheless remained substantially lower than RMD (0.93) and MS96 (0.75) on WGS in these 15 cancer types (S11 Fig). Finally, in the task of tumor subtyping the driver mutations were again less informative than passengers (S12A Fig), even though the difference is less striking than for cancer type classification. We have additionally tested classifiers that draw on refined OGM features that account for mutation impact, by: (i) thresholding or (ii) by weighting variants by CADD score [34], (iii) by using weighted pathway scores from the SAMBAR tool [35] and (iv) by restricting to the high-confidence set of driver mutations identified by Bailey *et al*. [33] (see Methods). Although this yielded slight gains in accuracy for some prioritization schemes, there were no significant overall improvements (best p = 0.11 for thresholding by CADD, S13 and S14 Figs). The OGM features had only modest accuracy in classifying tumors even after accounting for predicted mutation impact.

### Complementarity between passenger mutations features

Given that the two types of passenger mutation features–RMD and MS96 –appear quite informative in discriminating cancer types, we wondered if the information in them is redundant, or instead if each of them is uniquely proficient in classifying a particular set of tumor samples. The correlation between AUPRC scores of RMD and MS96 across the 18 tissues is modest (R^2^ = 0.24) suggesting that they might indeed be independently valuable in classifying tumors. For example, the RMD is more accurate in classifying colon, prostate and pancreatic cancer, while the MS96 is more proficient for kidney and medulloblastoma (S15 Fig).

Motivated by the above differences, we systematically evaluated whether the MS96 and the RMD are complementary in terms of individual tumor samples for which correct predictions can be obtained. To this end, we performed a classification with the RMD and the MS96 independently. Afterwards, we calculated how many of the misclassified tumor samples by the MS96 had been correctly classified by RMD and vice versa. Using the MS96 model, 468 samples out of 2264 were misclassified. Out of these 468, the majority (345) were correctly re-classified using RMD features (Fig 3B and 3C). On the other hand, starting with the RMD model, 287 samples out of 2264 were misclassified, and 164 out of the 287 samples could be correctly classified by applying the MS96 classifier (Fig 3B and 3C). We note that the two classifiers provided many complementary predictions even for tissues when overall accuracy was similar: for example, in uterine cancer RMD uniquely covered 17.6% of the predicted instances and MS96 20.9% of the instances (Fig 3B). This trend extends to cancer subtyping (S12B and S12C Fig).

**Fig 4:**
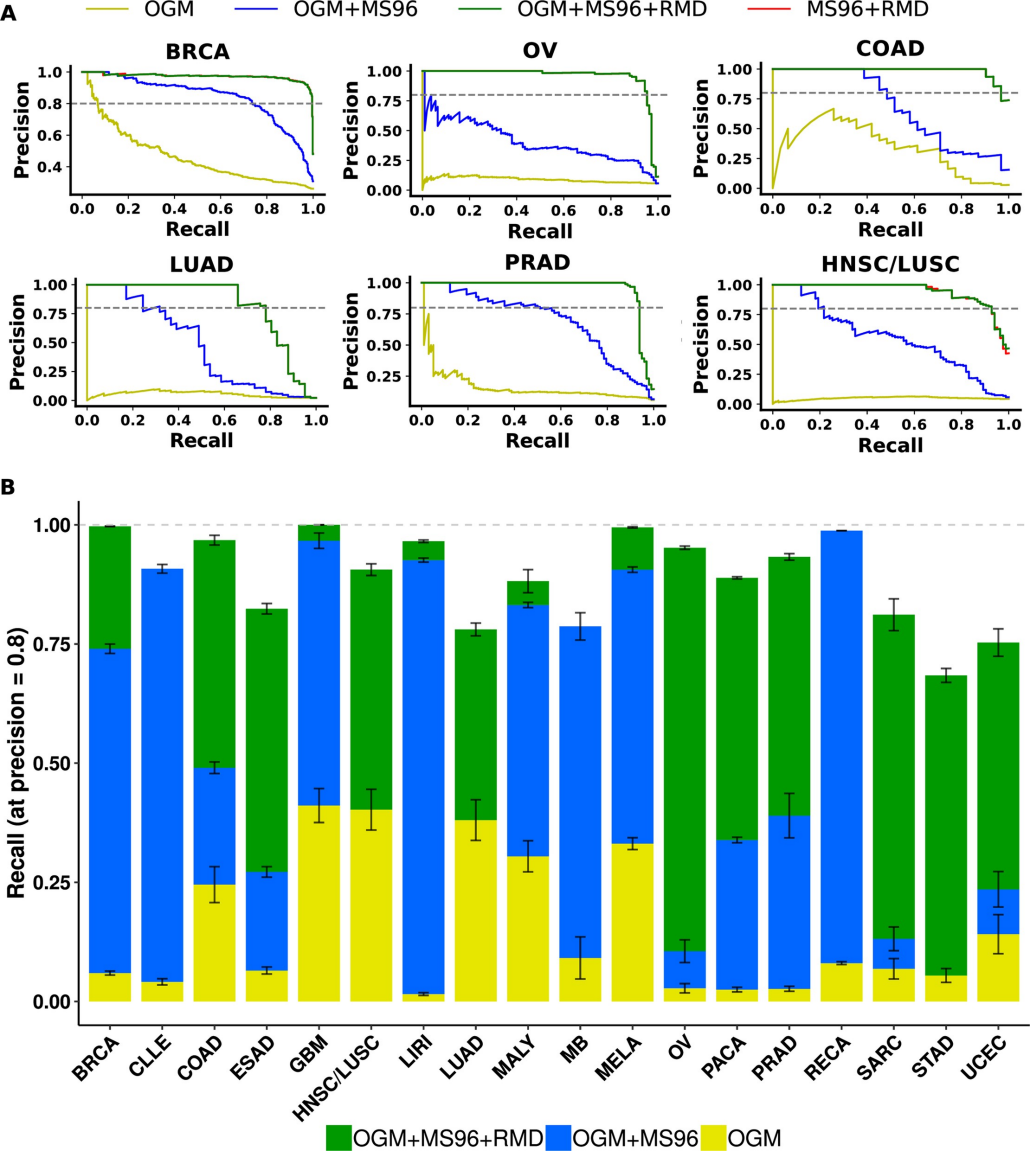
Combined classifiers provide substantially increased coverage with confident predictions. (A) Precision-Recall Curve for six example cancers (see S16 Fig for others) for the OGM driver mutations (yellow), for the combination of OGM and MS96 passenger mutations (blue), for the combination of MS96 and RMD (red) and for the combination of the OGM, MS96 and the RMD passenger mutations (green). Grey line indicates the threshold where precision = 0.8, equivalent to FDR = 20%. (B) Recall at FDR = 20% for the classification models trained on: OGM (yellow), OGM+MS96 (blue) and OGM+MS96+RMD features (green); height of the stacked bars indicates the excess proportion of patients receiving correct predictions upon introducing additional features to the classification model. See S17 and S18 Figs for the same analysis using only features from passenger mutations.

This complementarity suggests that a combination of both types of passenger mutation features would be beneficial for making a joint classification model. Thus, we compared the accuracy of a baseline OGM driver model to models drawing on both the drivers and the MS96 and RMD passengers, considered alone and in combination (Fig 4). As expected, the baseline and the combination models presented very different AUPRC scores of 0.21 ± 0.16 and 0.96 ± 0.11, respectively (median ± IQR across 18 cancer types, p<10^−15^, Wilcoxon test). To provide insight into practical implications of this observed increase in the AUPRC in the joint model, we compared the precision-recall curves for each cancer type (Fig 4A and S16 Fig) to estimate the number of cancer patients who would receive a confident diagnosis by the model after having introduced additional features. In particular, we estimated the *recall* score of the model–the proportion of all tumors of the relevant cancer type which was diagnosed–at the fixed *precision* of 0.8 (equivalent to false discovery rate, FDR = 20%) for that cancer type (grey line in Fig 4A and S16 Fig). Indeed for most cancer types a large increase in discovered cancer cases was enabled by use of the passenger features–first by introducing the MS96 and then by introducing both MS96 and RMD into a joint model. Notably, for ovarian cancer we observed an 8 percentage points (pp) increase in the number of patients correctly classified at FDR = 20% upon the addition of the MS96 (passengers) to the baseline OGM (drivers) and then a striking 85 pp increase upon further introducing the RMD passengers into the joint MS96+OGM model. Lung and esophagus adenocarcinoma, squamous cell carcinoma group (LUSC/HNSC), sarcoma, pancreatic, prostate, stomach and uterine cancer showed remarkable increases of ≥50 pp tumors discovered at FDR = 20% by including the RMD in addition to other features, while colorectal adenocarcinoma and breast cancer show an increase of approximately 40 and 10 pp, respectively, from use of RMD. Even cancers with good accuracy for the OGM+MS96 classifier, such as brain, lymphocytic leukemia, liver and melanoma, showed improvements by including the RMD (Fig 4B, S16 Fig and S5 Table), resulting in an overall gain of 50 pp (median over 18 cancer types) at FDR = 20% by use of RMD.

In summary, the passenger mutation features including the trinucleotide mutation spectra (MS96) and the domain-scale mutation rates (RMD) provide unique, non-redundant information for classifying individual tumors and should therefore be used in combination to provide coverage for a maximum number of patients.

### Mutation dropout and use of exome sequencing data

**Fig 5:**
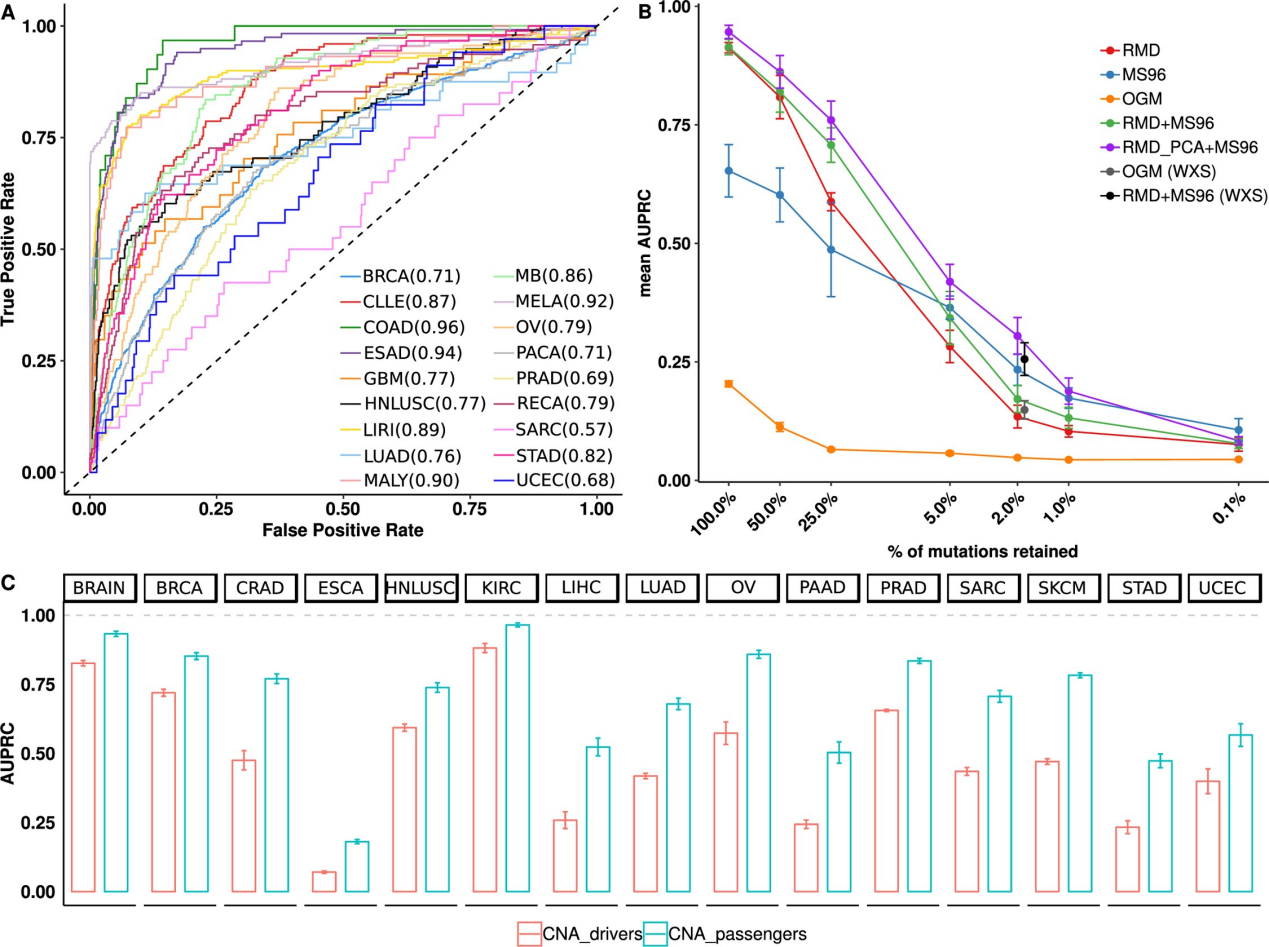
Predictive accuracy using data types other than whole-genome sequencing. (A) ROC curves for simulated whole-exome sequencing data, for one cancer type versus all others. Area under the ROC curve (mean across crossvalidation folds) for each cancer type is reported in the legend. (B) Crossvalidation AUPRC score for classifiers trained on various feature types under conditions of false negative mutation calls. (C) Crossvalidation AUPRC score for copy number states in 1Mb segments, estimated from SNP array data and tentatively divided into driver and passenger events. In (B) and (C), mean ± standard deviations are shown over multiple crossvalidation runs; (B) depicts the median score across all cancer types, and mean and s.d. thereof across crossvalidation runs are shown.

Whole-exome sequencing (WES) is becoming common among the diagnostic tests performed on cancer patients, offering cost savings over WGS while capturing most mutations known to be clinically relevant. Therefore, being able to predict cancer type of a metastatic tumor from this type of genomic data would be useful. Despite the fact that the exome only encompasses ~2% of the whole genome, the global passenger mutation features studied here (MS96 and RMD) are aggregate statistics across many genomic regions and thus have the potential to maintain the informative signal even with sparse data. Hence, we evaluated to which extent cancer type can be predicted from MS96 and the RMD features using exomes, by generating simulated WES data from the main WGS dataset and re-training the predictive models on such data. When using WES data, overall, a substantial loss of accuracy was observed for the passenger mutation features: only lymphoma, melanoma, colorectal and esophagus cancer show crossvalidation AUC≥0.9, and 10 of 18 cancer types dropped below AUC of 0.80 (Fig 5A).

Of note, the accuracy of the OGM driver features we used is generally not affected by moving from whole-genome to exome (there are minor differences due to low sequencing coverage in a certain number of exons; see Methods). This is because we consider only exonic coding mutations here, and additionally TERT promoter mutations, in accord with the latest estimates that non-coding cancer driver mutations appear to be rare [36]. Importantly, even this reduced accuracy of the passenger features (RMD and MS96) in WES is still higher than the accuracy of the driver mutations: 0.24 ± 0.35 (AUPRC median ± IQR across 18 cancer types) for RMD+MS96 passengers *versus* 0.17 ± 0.13 for OGM drivers in the simulated exomes. Therefore, for reduced representations of the genome, passenger mutation patterns still outperform drivers in classifying tumors. Finally, we evaluated the complementarity of the driver and the passenger features for the WES data and found that different classifiers are again able to classify the samples misclassified by the other method. In addition, greater proportions of tumors are uniquely correctly classified by passengers but not drivers, than by the drivers but not passengers (S19A Fig). Overall, we suggest that also for WES there is a tangible accuracy benefit from using combined classification models. In addition, we performed an external validation using the datasets from actual WES data from the TCGA consortium, while matching the cancer types to those we have in the main training dataset. For both datasets, the AUPRC scores were similar (S19B and S19C Fig) and showed no significant difference in a Wilcoxon test for the driver features (p = 0.27) nor the passenger features (p = 0.42).

An important application of genomic classifiers of cancer type and subtype would be to liquid biopsies, wherein tumoral DNA can be extracted at low purity (in many cases at <10% [14]). Moreover, in practice WGS is often applied at low coverage, for reasons of cost efficiency. Both of these factors result in the decreased ability to call somatic mutations, which adds noise and would affect the performance of genomic classifiers such as those we employ. Motivated by this, we tested the performance under conditions of false negative mutation calls (dropout), generating simulated genomes with 50% to 99.9% of all mutations removed at random. Expectedly, mutation dropout lowers performance of all classifiers, yet the passenger-mutation classifiers (RMD and MS96) (Fig 5B) are more robust. In particular, with 75% dropout, the RMD decreases 1.49-fold in its predictive performance (ratio of crossvalidation AUPRC), the MS96 decreases 1.24-fold, while driver mutations decrease 3-fold. This means that at this level the passenger mutations retain utility for classification (median AUPRC = 0.72 for RMD+MS96) while the drivers do not (median AUPRC = 0.07). At higher dropout (95%, 98%, 99% and 99.9% tested), the performance of all classifiers drops severely, but the relative advantage of passenger mutations is upheld (Fig 5B).

### Copy number alteration-based tumor type classifiers

In addition to analyzing the distribution of mutations, here implying single-nucleotide variants (SNVs) and small indels, we briefly examined the genomic distribution of copy-number alterations (CNA), motivated by the previous use of CNA data for tumor type classification [18,37]. Upon tentatively dividing CNA into driver and passenger events based on whether they affect a dosage-sensitive cancer gene (Supplementary Methods in S1 Text), we observed that in 15 of 15 tested cancer types, the global pattern of passenger CNA is more predictive than the 68 individual driver CNA (Fig 5C). Of note, passenger mutation patterns (RMD+MS96) are more predictive than either type of CNA feature. We acknowledge that it is difficult to disentangle driver from passenger CNA and that our data likely reflects a mix of the two types; see Supplementary Results in S1 Text.

## Discussion

There is a need for methods to detect tumor primary site, type and subtype in order to guide diagnostics and therapy. This is necessary for metastatic cancers of unknown primary (CUP) but also for screening the population-at-risk using emerging liquid biopsy techniques, wherein upon detection of tumoral DNA in bodily fluids, anatomical sites need to be prioritized. While there are established methods for tissue classification based on gene expression or DNA methylation in CUPs [11–13], genomic classifiers might provide an attractive alternative. This is because unlike the transcriptome and the epigenome, which diverge as the cells become malignant or when they metastasize to a new environment [38], the accumulated somatic mutations rarely revert. Moreover the tally of mutations arriving after tumorigenesis appears to be less numerous, compared to the mutations that had accumulated in the healthy cell before it turned cancerous [39]. In light of this, it is perhaps not surprising that the global patterns of passenger mutations, many arriving prior to cancer onset, accurately reflect the identity of the cell-of-origin. This is in contrast to the smaller number of driver mutations, which do affect cancer types differently to some extent but do not appear to possess sufficient information to distinguish the full diversity of cancers. In other words, mutational processes are more diverse between somatic cell types than are the selective pressures on oncogenic events, which tend to be shared across cancers.

A further attractive property of genomic classifiers is that mutations could plausibly be reliably detected in very impure samples–such as those originating from liquid biopsies–which might be challenging for extracting epigenomic or transcriptomic features. Given that approx. 30% of cancer patients were estimated to have enough circulating tumor DNA to make genome sequencing feasible [14], this opens the opportunity to use this genomic data for prioritizing cancer types, minimizing costly and invasive diagnostic tests and ensuring speedy access to therapy. Here, two important considerations are sequencing depth (coverage) and breadth (whole genomes *versus* exomes *versus* gene panels). Our simulation studies suggest that the passenger mutation patterns are to a certain extent robust to false negatives that might result from e.g. low sample purity or low-coverage sequencing, even though it remains to be established how the number and distribution of false negatives scales with reduced coverage and purity in realistic settings. The robustness stems from the fact that RMD and MS96 features do not rely strongly on detecting individual mutations, which could be missed in a particular patient, but instead draw on aggregate statistics that describe global distributions of mutation types. Therefore, methods based on genome-wide mutation patterns have the potential to generate models robust to noise in mutation calls, with applications to low-purity or low-coverage DNA sequencing.

Similarly, we find that also in case of exome sequences, the global mutation patterns derived from passengers outperform the driver mutations. Nevertheless accuracy is overall compromised, suggesting that WGS is superior to WES for tumor classification because it better captures the global patterns in passenger changes. By extrapolation, we expect what is relevant for WES to be even more so for gene panel sequencing, which has been gaining traction as a diagnostic tool to prioritize patients for therapies. The panels cover coding regions of ≤500 genes [40] and, given high sequencing coverage, have excellent ability to detect driver mutations. However, it is very difficult for a gene panel to capture a pattern of mutation rate variability across chromosomal domains (here, RMD), because many mutations in the panel will be subject to strong positive selection. Since panel sequencing provides only the driver features, it would therefore have a limited ability to classify cancer types (approximated by our OGM results), because it is blind to global mutation patterns emanating from passenger mutations. In addition, while it may be possible to infer driver CNA from panel sequencing data [41] thus boosting predictions of cancer type, the accuracy of such approaches remains to be established.

Our work highlights the surprising accuracy of the novel RMD features for classifying tumors. The tissue-specific signal in the global distribution of mutations across genomic domains was suggested to stem from differential replication timing programs in the cell-of-origin [26] or from differential chromatin accessibility [27]. Given how closely correlated these two variables are, causality still remains to be established, but the resulting mutation pattern nevertheless constitutes a very useful marker of the cell-of-origin. We currently use a simple representation for RMD: normalized mutation counts for each 1 Mb-sized genomic window. Given the reported correlation between neighboring and also distant 1Mb windows [26], we speculate that a simpler representation of RMD, more robust to noise, might be feasible. Indeed we tested a PC analysis on the RMD, as introduced previously [26], in the tissue classification task and found that it modestly increases accuracy (Fig 5B, label “RMD_PCA+MS96”), particularly for more noisy data resulting from false-negative mutation calls.

While the predictive power of RMD for cancer type, subtype and anatomical location is substantial, we suggest that RMD are best used in tandem with another type of global mutation pattern–the trinucleotide mutation spectrum (MS96)–since each provides predictions for distinct sets of tumor samples. This fits well with the current understanding of mechanisms that underlie these patterns: MS96 are thought to reflect the mutagenic exposures–either exogeneous like UV light [20], or endogeneous like defective DNA repair [42], which vary between cell types [21]. The RMD on the other hand describes the epigenome organization in the cell-of-origin [26,27]. This is reflected in the mutations that accumulate with cell divisions, even without necessitating a particular mutagenic exposure. The RMD thus provide a track record of the normal functioning of the cell, and MS96 reflect the extraordinary circumstances it has encountered on its road to cancer, describing different aspects of the natural history of each specific tumor.

Our machine learning classifiers using RMD, interestingly, made ocasional mis-classifications which appeared to be informative of the underlying cancer biology. This suggests the utility of RMD for defining novel cancer subtypes or refining existing ones, similary to how diverse omics data types reinforce and correct each other to yield robust molecular subtypes [6]. In this analysis, we have mainly focused in the application of RMD for identifying the tissue-of-origin of tumors, and known subtypes thereof. However, other clinical variables that are known to be associated with molecular subtypes–prognosis, propensity to metastasize and response to therapy–might be also possible to infer directly from RMD features, which remains as a direction for future work.

## Materials and methods

### Data collection and preparation

**Fig 6:**
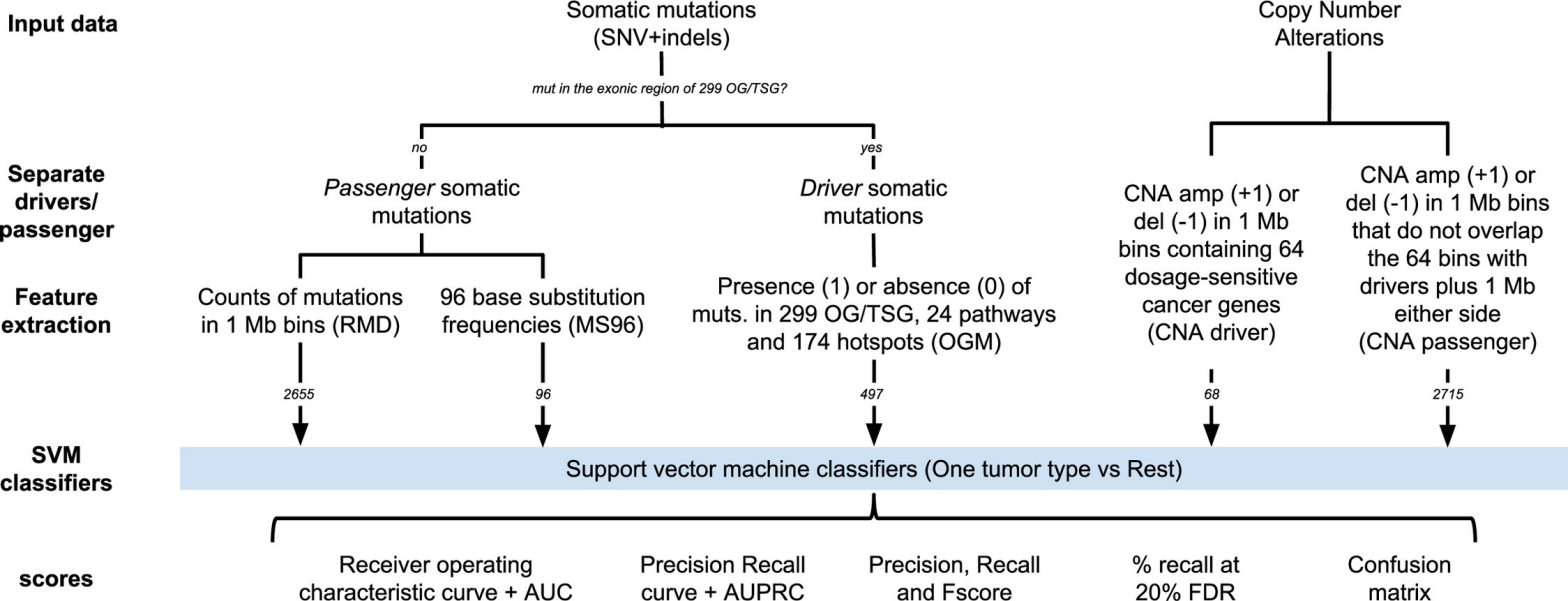
Schematic overview of the analyses.

We collated whole-genome sequencing (WGS) datasets of tumor somatic mutations from diverse sources to create a main training dataset containing cancer types with at least 20 tumor samples (Supplementary Methods in S1 Text and S1 Table). These were further subdivided into secondary training and validation sets with matching tumor types selected from different data sources or sequencing centers (S2 Table) and into subtypes based on molecular features and/or anatomical location (S3 Table). We masked out all regions in the genome defined in the ‘CRG Alignability 36’ track [43] as having imperfect mappability (<1.0), retaining 1.9 Gb of the *hg19* genome assembly. Additionally, we simulated whole-exome sequencing (WES) data from the WGS datasets by observing the actual sequencing coverage for TCGA exomes; details in Supplementary Methods in S1 Text. A schematic overview of the methodology is given in Fig 6.

### Genomic features calculation

To calculate regional mutation density (RMD) of passenger mutations, we filtered out all mutations in the coding regions of commonly mutated oncogenes (OG) and tumor suppressor genes (TSG) [33]. For each tumor, the RMD profile was calculated dividing each chromosome into 1 Mb windows and counting the number of mutations (SNVs and indels) in each window. Upon masking out regions of the genome with alignability <1.0, we normalized each count by dividing by the size of alignable regions (in Mb) in that window for WGS data, and by size of the capture regions (in Mb) for WES data (see below). We discarded 242 windows with less than 100 kb with alignability = 1 for WGS data, and 1472 windows with less than 10 kb covered by the capture regions for WES data. Chromosomes X and Y were excluded. The final RMD vector consisting of 2655 features (WGS) or 1597 (WES) was normalized by the average number of mutations per window in that tumor. We also considered reduced-redundancy versions of the WGS based features, by performing a k-medoids clustering (*pam* function in R) on the genomic windows and selecting 500 medoids, and moreover we performed a PCA (*prcomp* function in R) and selected the first 100 principal components.

We obtained the mutation spectrum for each tumor by calculating the relative frequencies of six mutation types (considered DNA strand-symmetrically: C>G, C>T, C>A, A>T, A>G, and A>C) in every trinucleotide context, yielding a set of 96 mutational spectrum (MS96) features. The mutations in 299 cancer driver genes were excluded from calculation.

To create the matrix of driver mutations we first examined the list of 299 significantly mutated OG and TSG [33], which we matched to a set of 1744 UCSC transcripts (using the UCSC Table browser) that were further examined for occurrence of mutations. For each sample, we generated a binary vector reflecting whether the exonic regions ±5 flanking nucleotides of any transcript of a given gene contains a somatic mutation (SNV or indel) or not. Secondly, we checked the presence of mutations in 24 cancer-related pathways [33]. For each sample, we generated a binary vector describing whether the exonic regions ±5nt of any of the genes in a particular pathway harbor a somatic mutation (SNV or indel). Thirdly, we checked the presence of mutations in individual hotspots. We downloaded ‘All Mutations in Census Genes’ dataset from COSMIC [44] and selected as hotspot positions the coordinates where mutations were observed at least 100 times ±5nt. For each sample, we generated a binary vector describing whether each hotspot position was mutated, yielding 174 features. Finally, we concatenated the three binary matrices.

In addition, we generated six additional sets of refined OGM features to look into whether prioritizing the mutations according to functional impact improves ability to classify cancer type. First, to adjust for the influence of the overall tumor mutation burden and of gene length on the occurrence of mutations in cancer genes, we substituted the original pathway features for those obtained from the SAMBAR tool [35]. Secondly, to account for the functional impact of mutations, we stratified the mutations into putatively high and low impact using CADD scores [34], and created the OGM features only using putative high-impact mutations while ignoring the low-impact mutations, using two different stringency thresholds (>10 and >20; a mutation with a CADD score of 10 means that it is among the top 10% most impactful variants, and score of 20 top 1%). We additionally tested using CADD score directly as a weight, replacing the 0/1 indicator in the input data matrix. Thirdly, we generate the OGM features using only the ~3400 driver mutations reported by Bailey *et al*. [33] and additionally a set of features that represents each of these driver mutation loci individually as a feature (1 if mutation present, 0 if absent).

### Training predictive models

For cancer type classification, we generated one model per cancer type to discriminate it from the rest of tumors of other cancer types pooled together (one-vs-rest). For training the model and assessing its performance, we used the models and functions in the *sklearn* library [45].

#### Machine learning algorithms

We applied the Support Vector Machine (SVM) algorithm for classification [46]. Briefly, SVM is a supervised machine learning method that searches for the hyperplane that maximizes the width of a margin between the instances of opposite classes. We used the SVC function of *sklearn*.*svm*, combined with OneVsRestClassifier function from *sklearn*.*multiclass* module. To account for the class imbalance, we introduced class weights, calculated as the total number of samples divided by the number of classes multiplied by the number of samples of a class. In addition, the metaparameters of the SVM radial basis function kernel, *C* and *gamma*, were optimized by a grid search to maximize crossvalidation accuracy as recommended in the LibSVM best practices [47], for each training set separately. In addition, we applied the RandomForestClassifier function as implemented in *sklearn*.*ensemble*, where no hyperparameters were optimized and a forest size of 500 trees was trained.

#### Evaluating model accuracy

The performance of the model was assessed for each method by calculating the ROC curve and the Area Under the ROC Curve (AUC) for each cancer type using five-fold crossvalidation. Each AUC score was obtained in 10 runs of crossvalidation, and the mean, median, standard deviation (sd) and interquartile range (IQR) thereof were calculated. Similarly, we calculated the precision-recall (P-R) curve and the Area Under the P-R Curve (AUPRC) for each cancer type using five-fold crossvalidation. In addition, the precision, recall and F-score values were obtained 10 times and the previous statistics were calculated. Additionally, to quantify the improvement of the classification accuracy by introducing additional sets of features into a joint model, we calculated the precision-recall curve with five-fold cross validation, repeated 10 times. We determined the recall score at the precision = 0.8 (equivalent to 20% FDR) for the classifiers derived from OGM, from OGM+MS96, from OGM+MS96+RMD and from RMD+MS96 features. Precision and recall are TP/(TP+FP) and TP/(TP+FN), respectively, where TP is the number of true positives, FP false positives and FN false negatives. The mean and standard deviation of recall scores across crossvalidation runs were determined. The difference in recall can be interpreted as the percentage point (pp) increase of correctly classified patients due to the addition of a new set of features to the baseline set of features (OGM). In addition to crossvalidation, we also provide results on independent genomic data sets, which originate from a different study and/or sequencing center (see S2 and S4 Tables).

#### Feature importance calculation

We generated a list of most the informative features obtained by four different supervised feature selection methods. These are (i) Elastic Net regularized regression, (ii) Random Forest feature importance, (iii) the Relief-F method and (iv) Information Gain, which we applied to RMD classifiers and to OGM+RMD+MS96 classifiers for each cancer type. For the RMD classifiers, we reported the RMD features that were found to be in the top 25 consistently by two or more different methods. Additionally, for the OGM+RMD+MS96 classifiers we reported the top 25 features for each set of features in each cancer type, along with their rank within the combined classifier. Furthermore, we applied feature selection to the classifier during crossvalidation to examine effects of reduced feature sets on accuracy of models. For this, in each fold of the crossvalidation we fit an Elastic Net model in the training set and select the features whose coefficients in the EN were different from zero.

## Supporting information

S1 Fig Summary statistics of the main training dataset. (A) Distribution of the total number of mutations per sample for each cancer type. (B) Precision, Recall and F-score values for each cancer type. Each bar represents the mean of the corresponding score obtained from five independent runs (5-fold cross validation in each run) for each cancer type. Error bars represent the standard error of the mean for each cancer type.
(PDF)

S2 Fig Analysis of possible batch effects: Sequencing center. Biplots for combinations of the 5 first principal components (PCs) for the RMD features, in a subset of cancer types with at least 5 samples per annotation group. Scree plot shows variance explained by each PC. The above-diagonal part of the scatterplot is colored by cancer type. The below-diagonal part of the scatterplot is colored by sequencing center.
(PDF)

S3 Fig Analysis of possible batch effects: Age. Biplots for combinations of the 5 first principal components (PCs) for the RMD features, in a subset of cancer types with at least 5 samples per annotation group. Scree plot shows the variance explained by each PC. The above-diagonal part of the scatterplot is colored by cancer type. The below-diagonal part of the scatterplot is colored by age (patients stratified in old and young groups according to whether their age is greater or lower than the median for each cancer type respectively).
(PDF)

S4 Fig Analysis of possible batch effects: Ancestry. Biplots for combinations of the first 5 principal components (PCs) for the RMD features, in a subset of cancer types with at least 5 samples per annotation group. Scree plot shows the variance explained by each PC. The above-diagonal part of the scatterplot is colored by cancer type. The below-diagonal part of the scatterplot is colored by the ancestry group of the patient.
(PDF)

S5 Fig Analysis of possible batch effects: Ancestry and age. (A) Area Under the Precision Recall curve (AUPRC) for RMD features in a subset of three cancer types training in samples from one group (european) and testing on another group (pooled asian, african and Hawaiian) in red; and training and testing in a mixture of groups maintaining the proportions between training and testing sets in blue. (B) AUPRC for RMD features in a subset of 3 cancer types training in samples from one group (old—age above the median of the cancer type) and testing on another group (young—age below the median of the cancer type) in red; and training and testing in a mixture of groups maintaining the proportions between training and testing sets in blue.
(PDF)

S6 Fig Description of the secondary training and external validation datasets. (A) Distribution of the total number of mutations per sample for each cancer type of the secondary training dataset (red) and the external validation dataset (blue). (B) For RMD features AUC mean of 5 independent runs obtained by training in the secondary training dataset and testing in the external validation dataset (blue) and by crossvalidation in the secondary training dataset (red) for each cancer type. Error bars represents the standard error of the mean of each cancer type. (C) For RMD features AUPRC mean of 5 independent runs obtained by training in the secondary training dataset and testing in the external validation dataset (blue) and by crossvalidation in the secondary training dataset (red) for each cancer type. Error bars represents the standard error of the mean of each cancer type.
(PDF)

S7 Fig Evaluation of RMD with randomized labels. Area Under the ROC curve (AUC) for each cancer type calculated with RMD classifiers with the class labels (cancer type) randomized. Each dot corresponds to one independent run (10 runs in total).
(PDF)

S8 Fig Evaluation of subtype classifiers performance. Receiver Operating Characteristic (ROC) curve for each subtype versus the rest of samples, in breast cancer (A), colorectal cancer (B), head and neck squamous cell adenocarcinoma (C), liver cancer (D), melanoma (E) and stomach cancer (F) datasets. Area Under the ROC curve (AUC) reported (mean and standard error of the mean across 5-fold cross validation rounds) in the legend of each panel.
(PDF)

S9 Fig Evaluation of predictive accuracy of RMD features after dimensionality reduction. (A) Mean Area Under the ROC Curve (AUC) for each cancer type for the full set regional mutation density (RMD) features in yellow and the reduced set of 500 RMD features (by k-medoid clustering) in blue. (B) Mean Area Under the Precision Recall Curve (AUPRC) for each cancer type for the full set of RMD features in yellow and a reduced set of 500 RMD features (by k-medoid clustering) in blue. Error bars are the standard error of the mean.
(PDF)

S10 Fig Evaluation of RMD with and without feature selection. Area Under the Precision Recall Curve (AUPRC) for the main training dataset for each cancer type with all RMD features (in green); and applying feature selection to each training subset using Elastic Net and testing with those selected features within each fold of the crossvalidation (in orange). Each dot represents one cancer types (median AUPRC across the 3 folds of the crossvalidation, distinguishing that cancer type versus the rest of cancer types).
(PDF)

S11 Fig Evaluation of model accuracy using passenger *versus* driver mutation features. Mean Area Under the Precision Recall Curve (AUPRC) scores for each cancer type in the main training dataset, using regional mutation density (RMD) features in red, and 96 mutation spectra (MS96) features in green. The presence/absence of mutations in cancer genes (OGM features) was determined for a dataset of real WES with equivalent cancer types (shown in blue).
(PDF)

S12 Fig Comparison and complementarity analysis for subtypes using RMD features. (A) Mean Area Under the Precision Recall Curve (AUPRC) scores for each cancer type for the subtypes of 6 major cancer types, using different sets of features: regional mutation density (RMD) in red, 96 mutation spectra (MS96) in green and presence/absence of oncogenic mutations (OGM) in blue. (B) For the subtypes datasets of six cancer types % of samples that are: (i) correctly classified by both RMD and MS96 (yellow), (ii) misclassified by both methods (gray), (iii) correctly classified by the MS96 but not by the RMD (blue) and (iv) correctly classified by the RMD but not by the MS96 (red). (C) Venn diagram of samples correctly classified by MS96, RMD or OGM features and their intersections for the subtypes classification.
(PDF)

S13 Fig Evaluation of features that account for mutation impact. (A) Area Under the Precision Recall curve (AUPRC) for five sets of oncogenic mutation (OGM) features (see *Methods*) on WGS data. P-value reported for each set of features compares with the default OGM features as a baseline, using one-tailed Wilcoxon signed rank test (with alternative set to “less” in the R function *wilcox*.*test*). (B) Area Under the Precision Recall curve (AUPRC) for three different sets of OGM features (see *Methods*) in a subset of 560 patients (only considering genomes with at least one mutation from the Bailey *et al*. list). P-values for each set of features compare with the default OGM features as a baseline one, using a statistical test as in (A).
(PDF)

S14 Fig Evaluation of features that account for mutation impact. (A) Area Under the Precision Recall curve (AUPRC) for five sets of oncogenic mutation (OGM) features (see *Methods*) on WES data. P-value reported for each set of features compares against the baseline OGM features using a Wilcoxon signed rank test, one-tailed (alternative set to “less” in the R function *wilcox*.*test*).
(PDF)

S15 Fig Comparison of accuracy of RMD and MS96 features per cancer type. Mean Area Under the Precision Recall curve (AUPRC) score of the RMD features (x axis) versus MS96 features (y axis) of the main training dataset, in crossvalidation. Error bars are the standard deviation of AUPRC for RMD features (x axis) and MS96 features (y axis).
(PDF)

S16 Fig Gains in classifier accuracy by introducing additional sets of features. Precision-Recall Curve for each cancer type for the OGM (yellow), for the combination of OGM and MS96 (blue) and for the combination of the OGM, MS96 and the RMD (green), and MS96 and RMD without OGM (red) features for the main training dataset. In most cancer types the red curve overlaps the green curve perfectly and is thus hidden on the plots. Grey line indicates the threshold where precision = 0.8.
(PDF)

S17 Fig Improvement in accuracy of RMD-based classifiers by the addition of MS96 features. Precision-Recall Curve for each cancer type for the RMD (blue) and for the combination of RMD and MS96 (purple) for the main training dataset. Grey line indicates the threshold where precision is equal to 0.8
(PDF)

S18 Fig Improvement of RMD classifiers by the addition of MS96 features. Recall at FDR = 20% for the classification models trained on RMD (blue) and RMD+MS96 features (purple); height of the stacked bars indicates the excess proportion of patients receiving correct predictions upon introducing the additional features to the classification model. Bars show the mean of five cross-validation runs, and error bars are standard deviations.
(PDF)

S19 Fig Complementarity and external validation of features with simulated WES datasets. (A) For the main training dataset of simulated WES, fraction of samples that are: (1) correctly classified by both the passengers (RMD+MS96) and the drivers (OGM) (yellow), (2) misclassified by both methods (gray), (3) correctly classified by the passengers but not by the drivers (orange) and (4) correctly classified by the drivers but not by the passengers (green). (B) For RMD+MS96 features dataset, mean AUPRC of five classification runs obtained by training on the simulated WES dataset and testing on the real WES dataset as an external validation (blue) and by crossvalidation in the secondary training dataset (red) for each cancer type. Error bars represents the standard error of the mean of each cancer type. (C) As above, but for OGM features. Error bars represents the standard error of the mean of each cancer type.
(PDF)

S1 Table Description of the datasets that form the main training dataset. (XLSX)

S2 Table Paired datasets from different sources or sequencing centers for external validation. In column 1 the cancer type, in column 2 and 3, the dataset name, the number of samples in brackets and the source.
(XLSX)

S3 Table Description of the subtypes of the six major cancer types and their source. (XLSX)

S4 Table Description of the data used for simulated WES and real WES from TCGA. (XLSX)

S5 Table Comparison of AUC score for secondary training dataset in crossvalidation (AUC crossvalidation) and AUC score training in secondary training dataset and testing in External validation dataset (AUC external validation). Refer to S2 Table for a detail description of each dataset.
(XLSX)

S6 Table Mean Recall at precision 0.8 for different combination of features for the main training dataset. (XLSX)

S7 Table Most informative features for tissue classification. (A) Top 25 features ranked by 4 different suppervised feature selection methods for RMD classifiers for each cancer type. (B) Top 25 features ranked by 4 different suppervised feature selection methods for RMD classifiers for each subtype (separate classifiers for each cancer type). (C) Top 25 features ranked by 4 different suppervised feature selection methods for RMD+MS96+OGM classifiers for each cancer type. In each tab the top 25 features for each set of features are reported, along with their rank in the combined classifier.
(XLSX)

S1 Text Methods: Collection and preparation of genomic data, obtaining copy number alteration data. (PDF)
