# Supplementary figures and images for: Passenger mutations accurately classify human tumors
Source: PLoS Comput Biol. 2019 Apr 15;15(4):e1006953. doi: 10.1371/journal.pcbi.1006953 (PMC6483366; doi:10.1371/journal.pcbi.1006953)

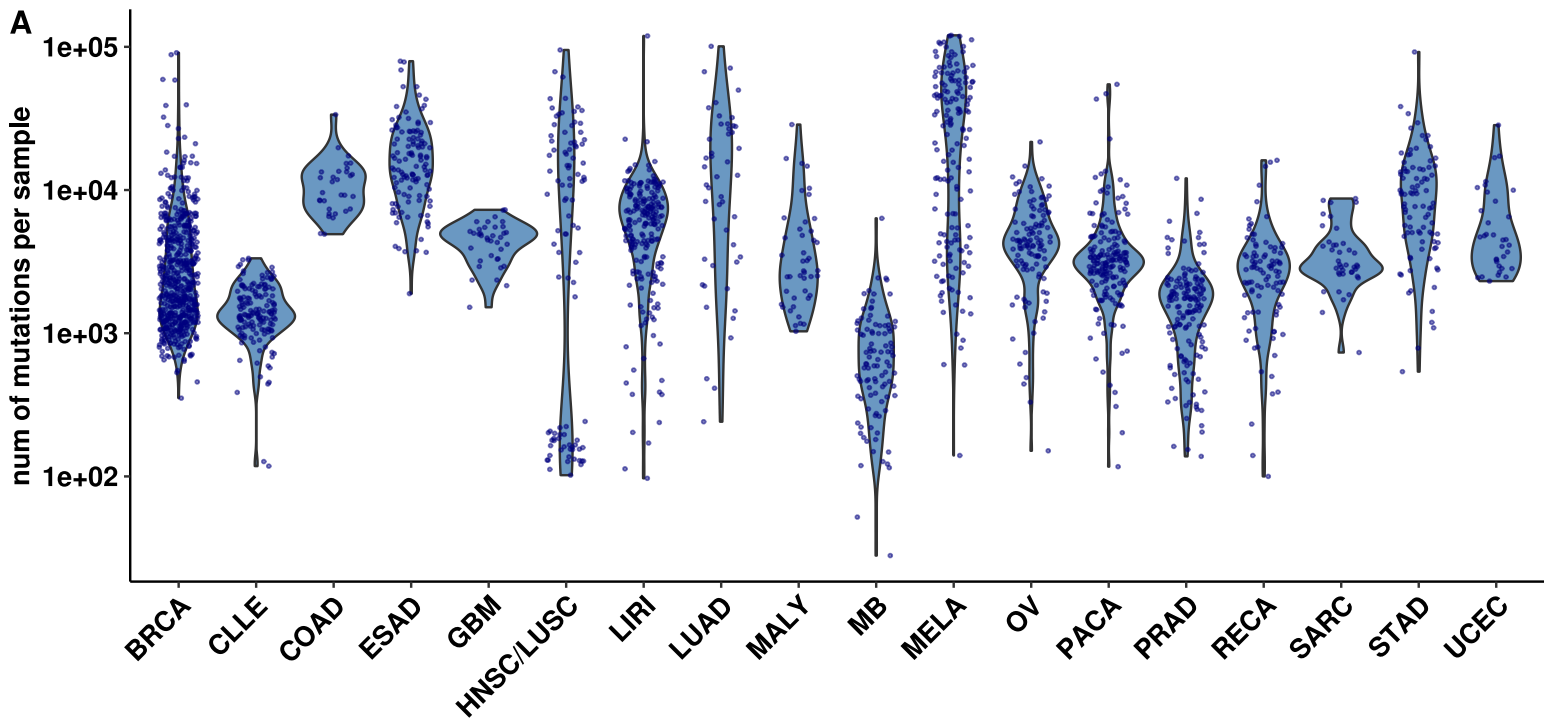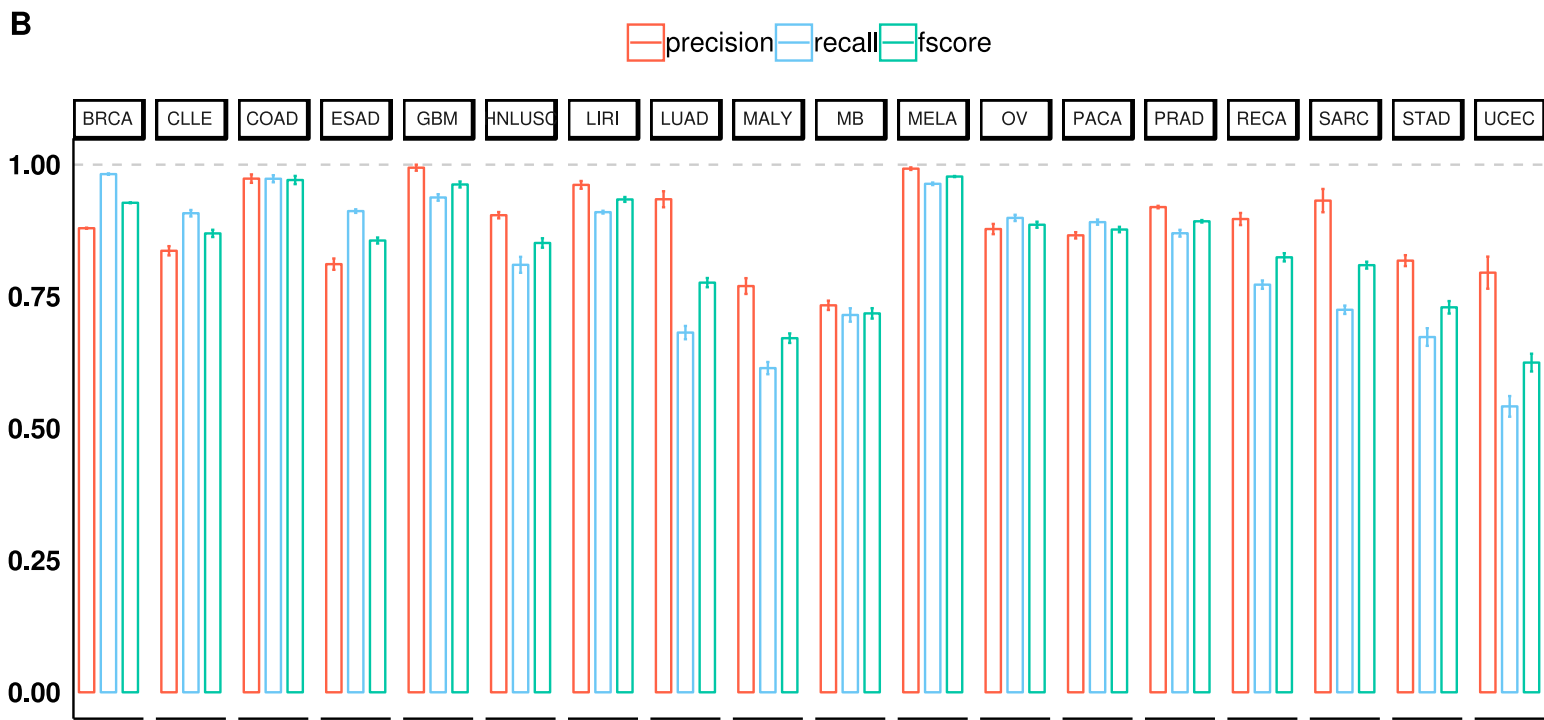

Supplement: S1 Fig — (A) Distribution of the total number of mutations per sample for each cancer type. (B) Precision, Recall and F-score values for each cancer type. Each bar represents the mean of the corresponding score obtained from five independent runs (5-fold cross validation in each run) for each cancer type. Error bars represent the standard error of the mean for each cancer type. (PDF) [file pcbi.1006953.s001.pdf]

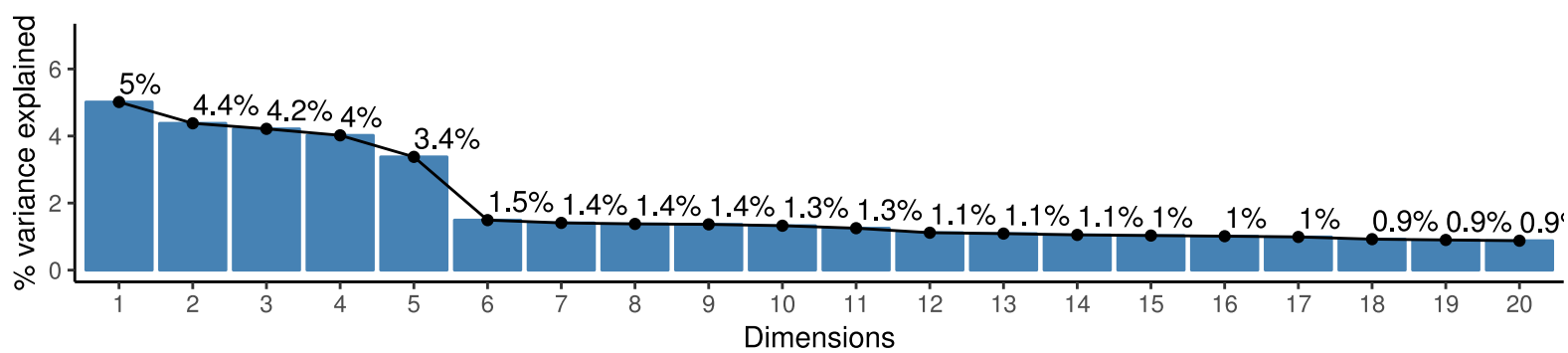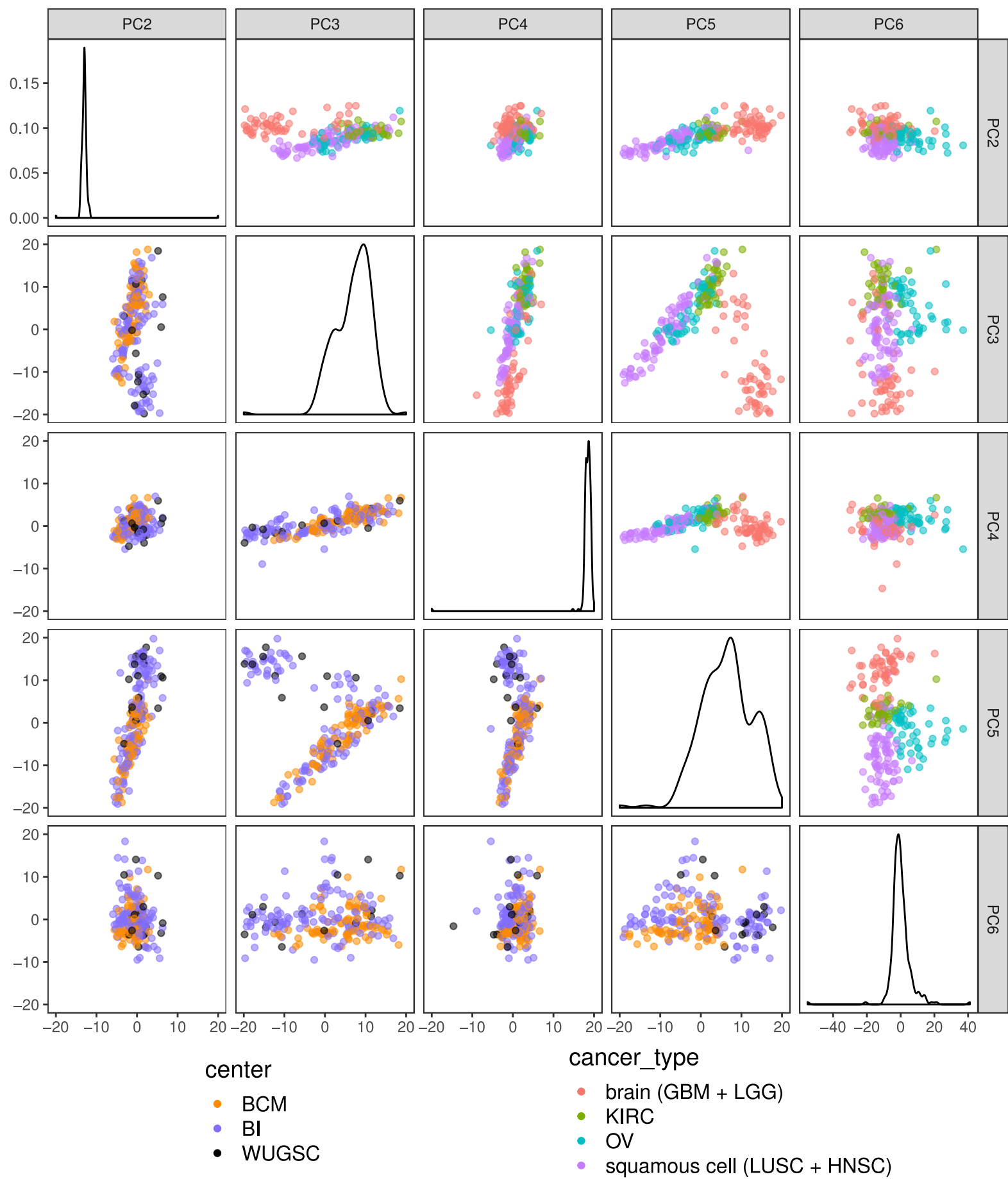

Supplement: S2 Fig — Biplots for combinations of the 5 first principal components (PCs) for the RMD features, in a subset of cancer types with at least 5 samples per annotation group. Scree plot shows variance explained by each PC. The above-diagonal part of the scatterplot is colored by cancer type. The below-diagonal part of the scatterplot is colored by sequencing center. (PDF) [file pcbi.1006953.s002.pdf]

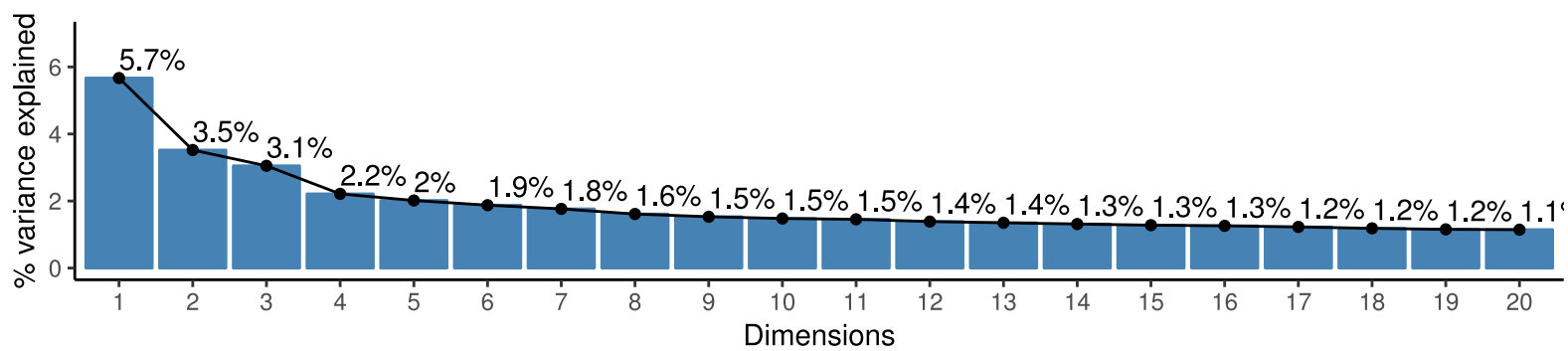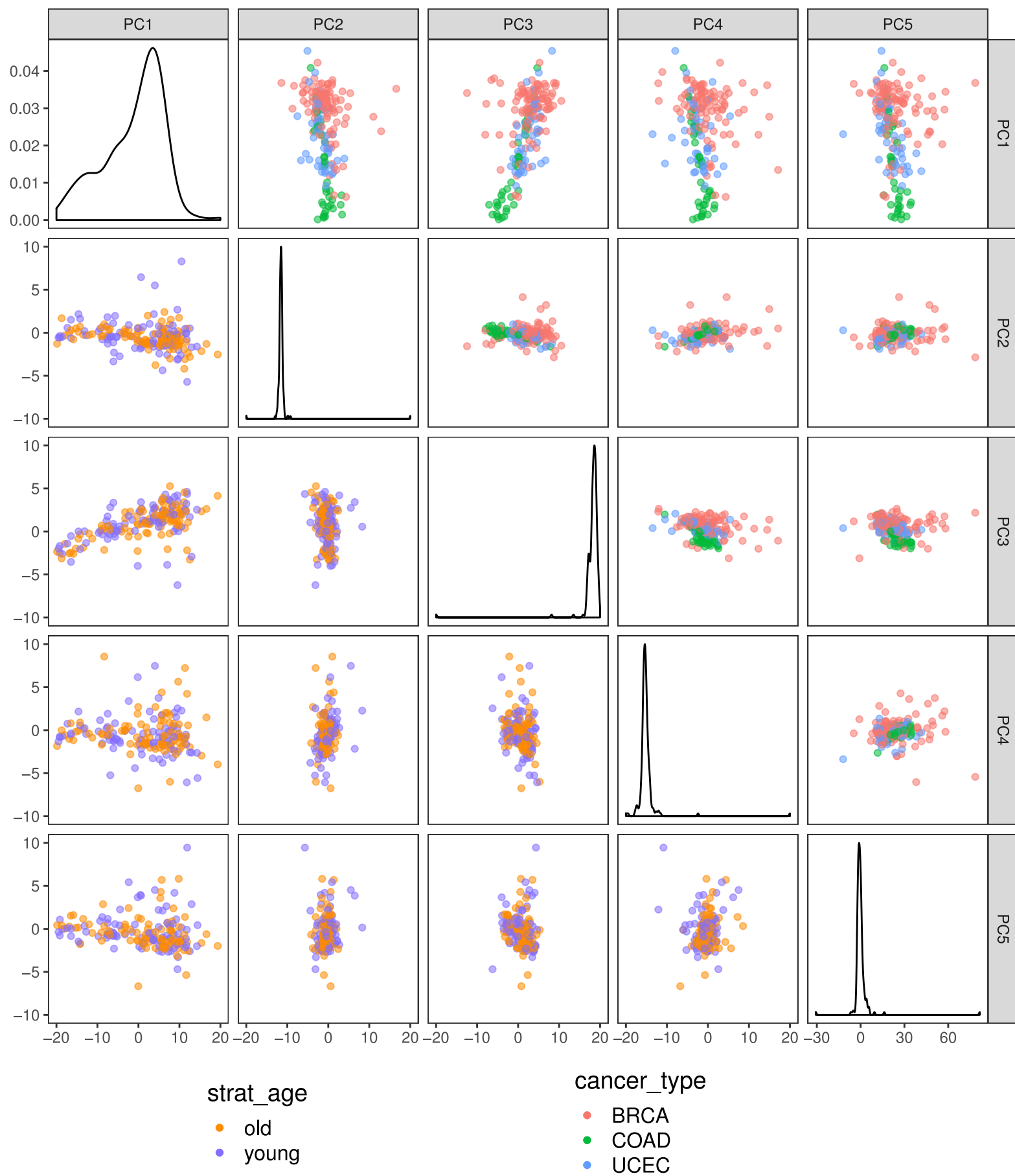

Supplement: S3 Fig — Biplots for combinations of the 5 first principal components (PCs) for the RMD features, in a subset of cancer types with at least 5 samples per annotation group. Scree plot shows the variance explained by each PC. The above-diagonal part of the scatterplot is colored by cancer type. The below-diagonal part of the scatterplot is colored by age (patients stratified in old and young groups according to whether their age is greater or lower than the median for each cancer type respectively). (PDF) [file pcbi.1006953.s003.pdf]

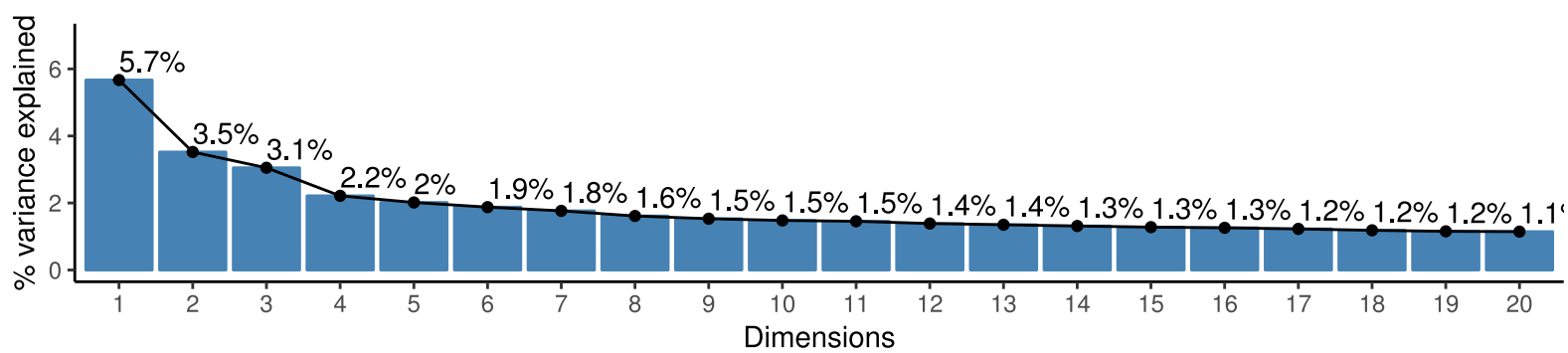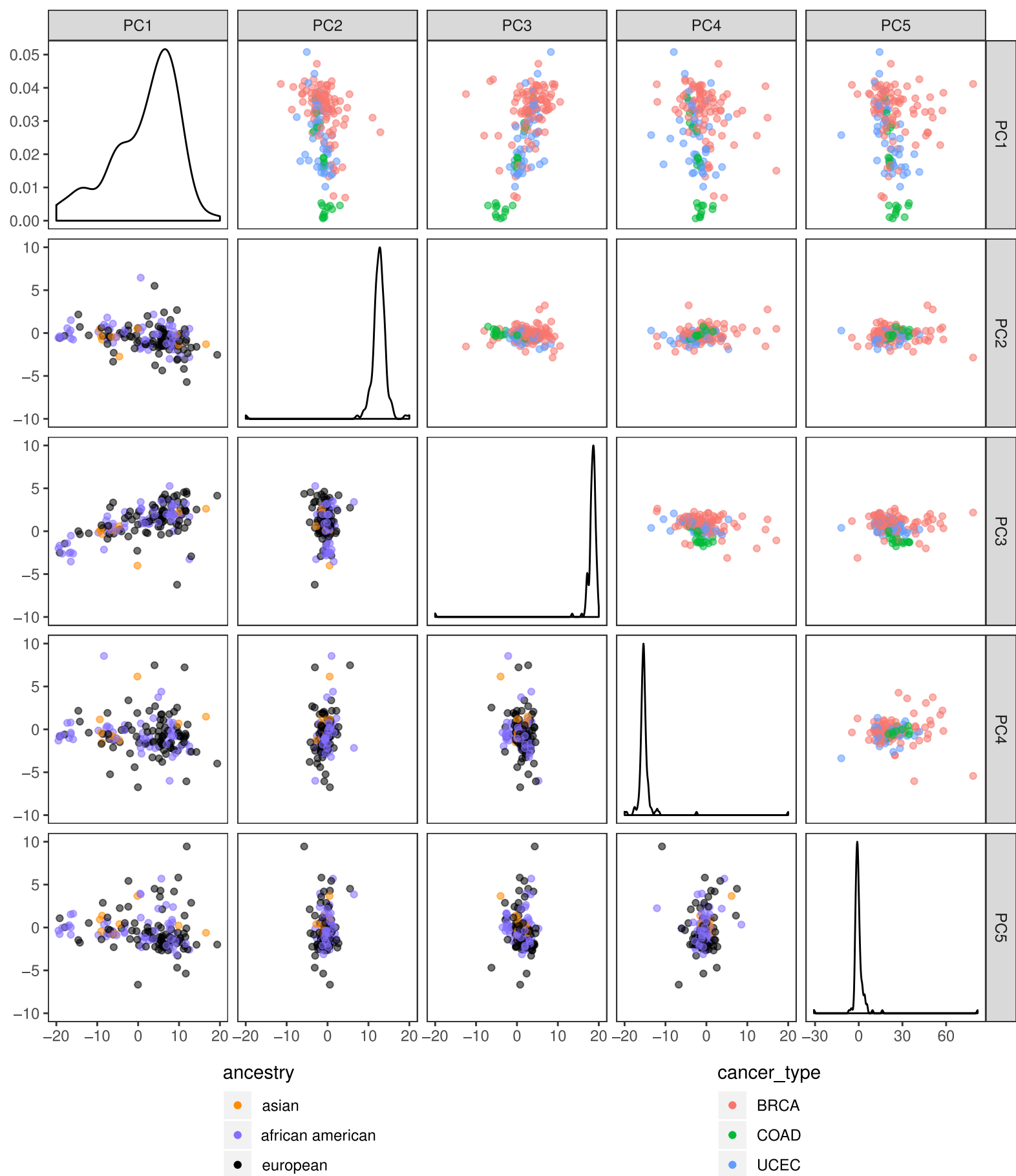

Supplement: S4 Fig — Biplots for combinations of the first 5 principal components (PCs) for the RMD features, in a subset of cancer types with at least 5 samples per annotation group. Scree plot shows the variance explained by each PC. The above-diagonal part of the scatterplot is colored by cancer type. The below-diagonal part of the scatterplot is colored by the ancestry group of the patient. (PDF) [file pcbi.1006953.s004.pdf]

**A**

RMD\_race RMD\_race\_RANDOMsamp

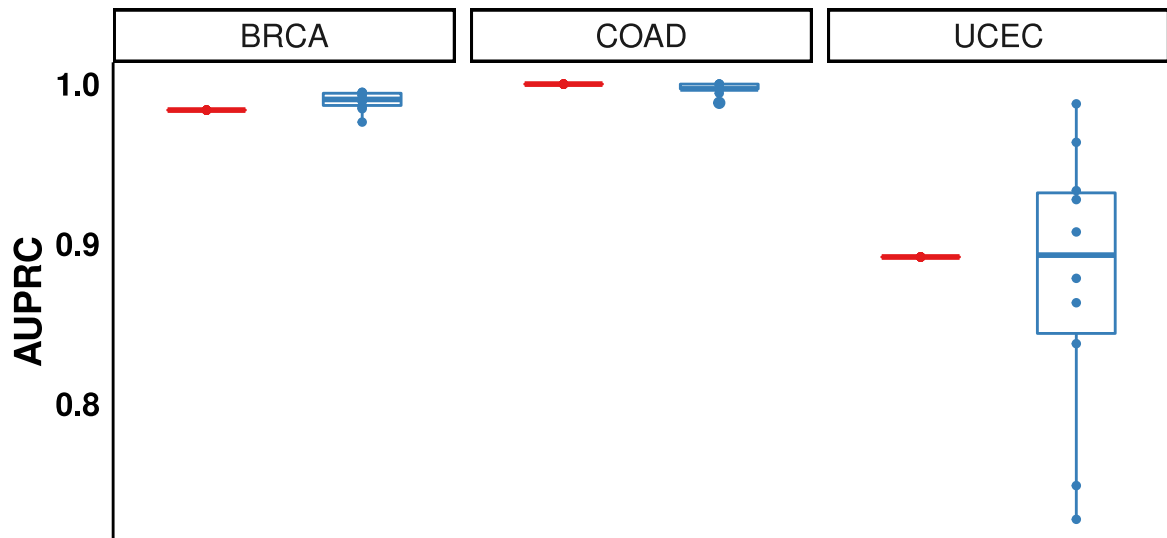**B**

RMD\_age RMD\_age\_RANDOMsamp

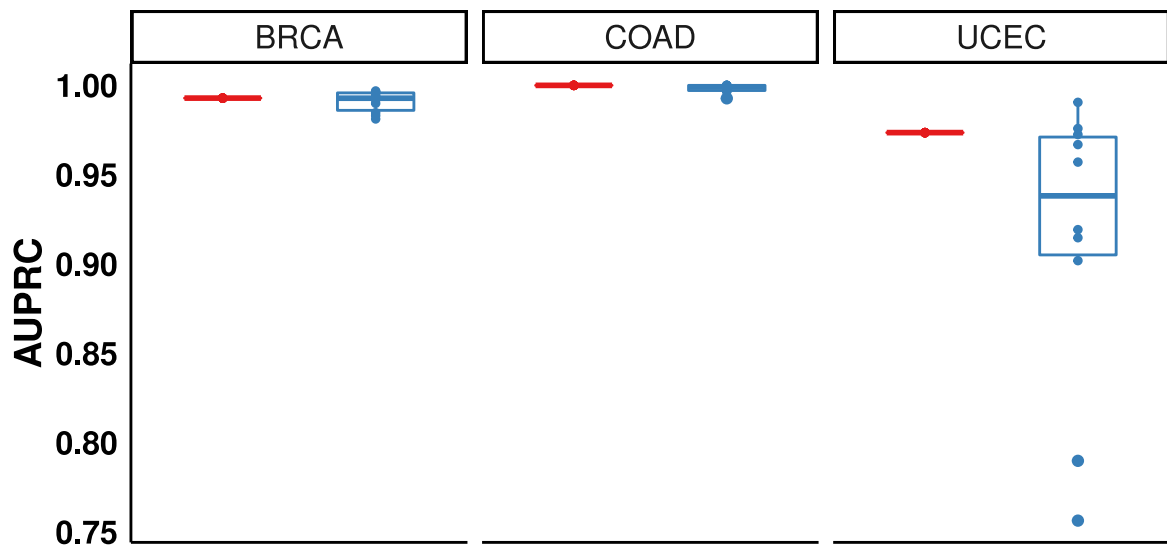

Supplement: S5 Fig — (A) Area Under the Precision Recall curve (AUPRC) for RMD features in a subset of three cancer types training in samples from one group (european) and testing on another group (pooled asian, african and Hawaiian) in red; and training and testing in a mixture of groups maintaining the proportions between training and testing sets in blue. (B) AUPRC for RMD features in a subset of 3 cancer types training in samples from one group (old—age above the median of the cancer type) and testing on another group (young—age below the median of the cancer type) in red; and training and testing in a mixture of groups maintaining the proportions between training and testing sets in blue. (PDF) [file pcbi.1006953.s005.pdf]

**A**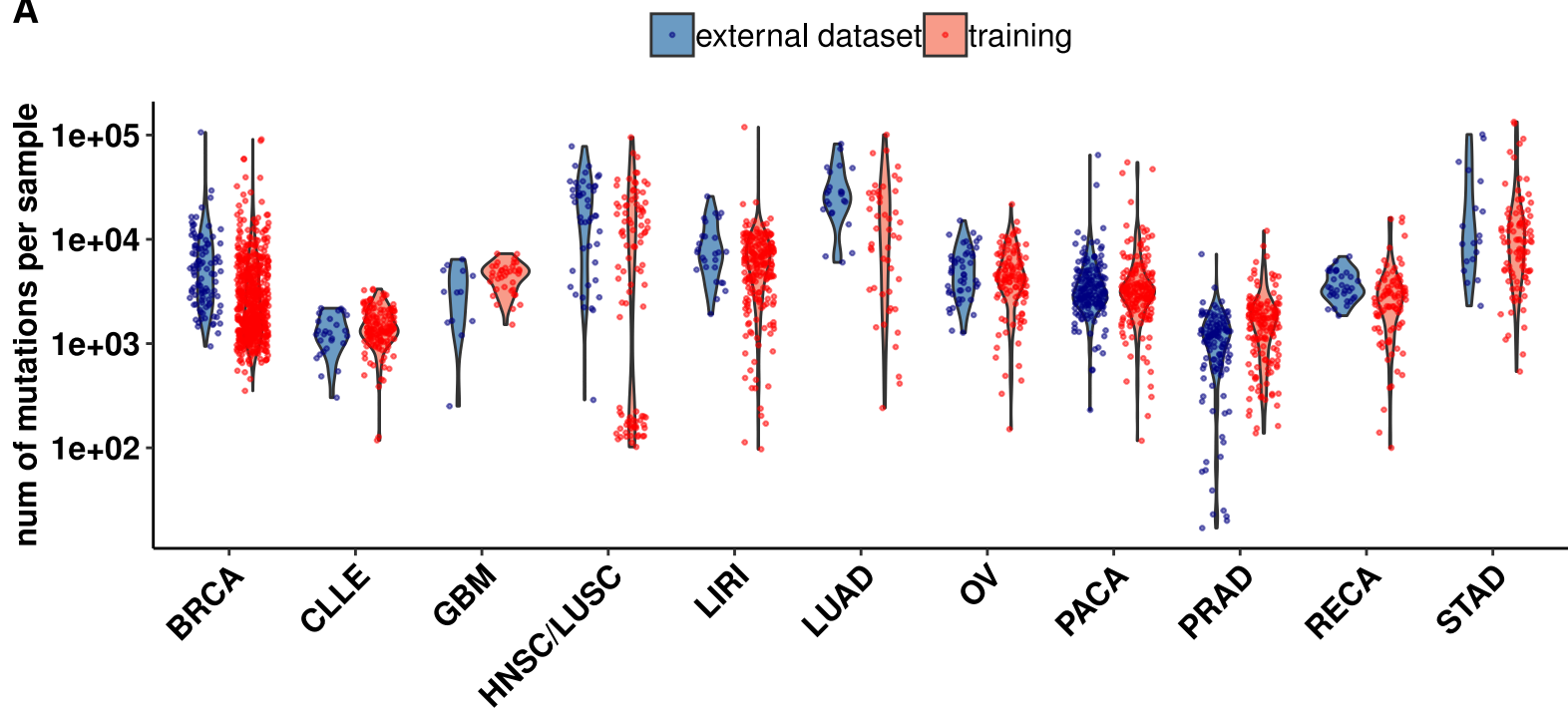**B**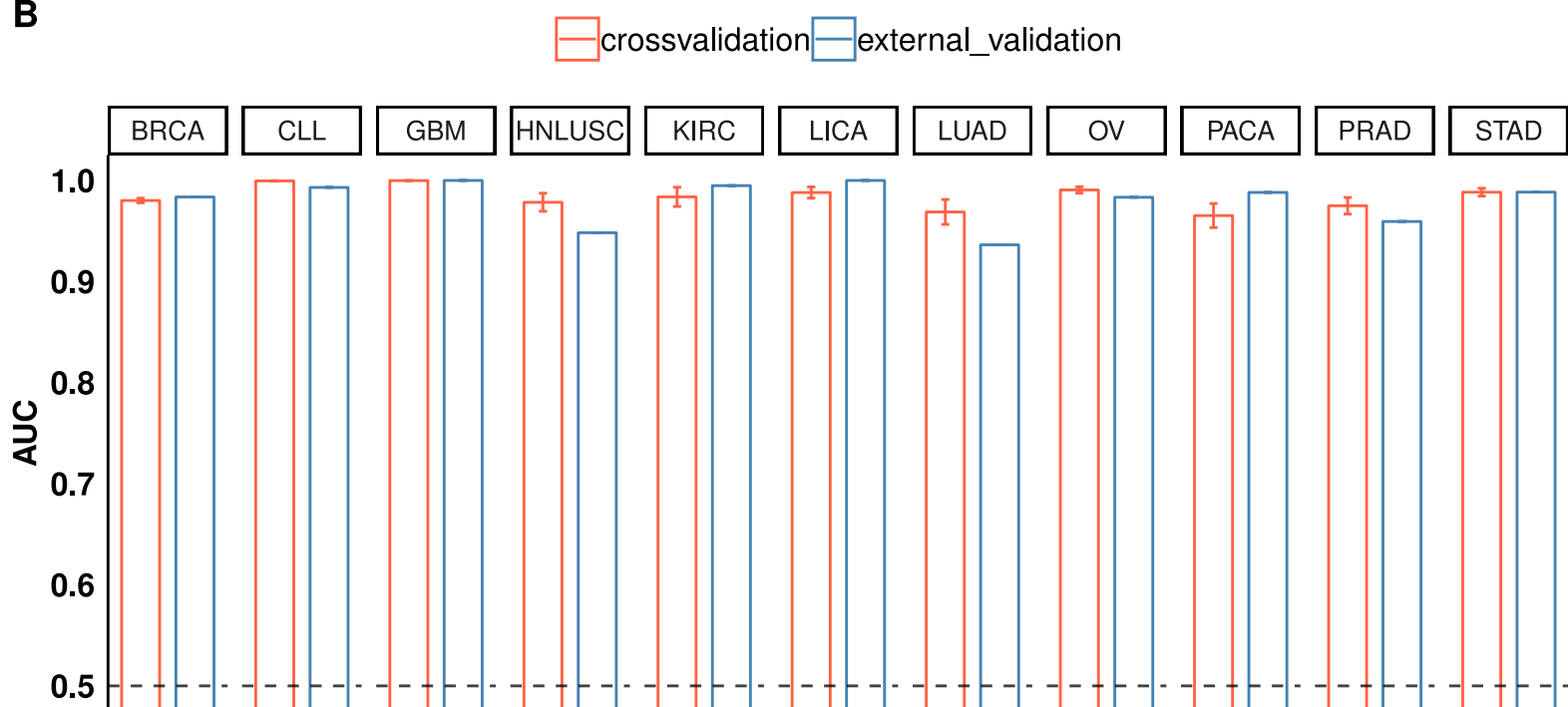**C**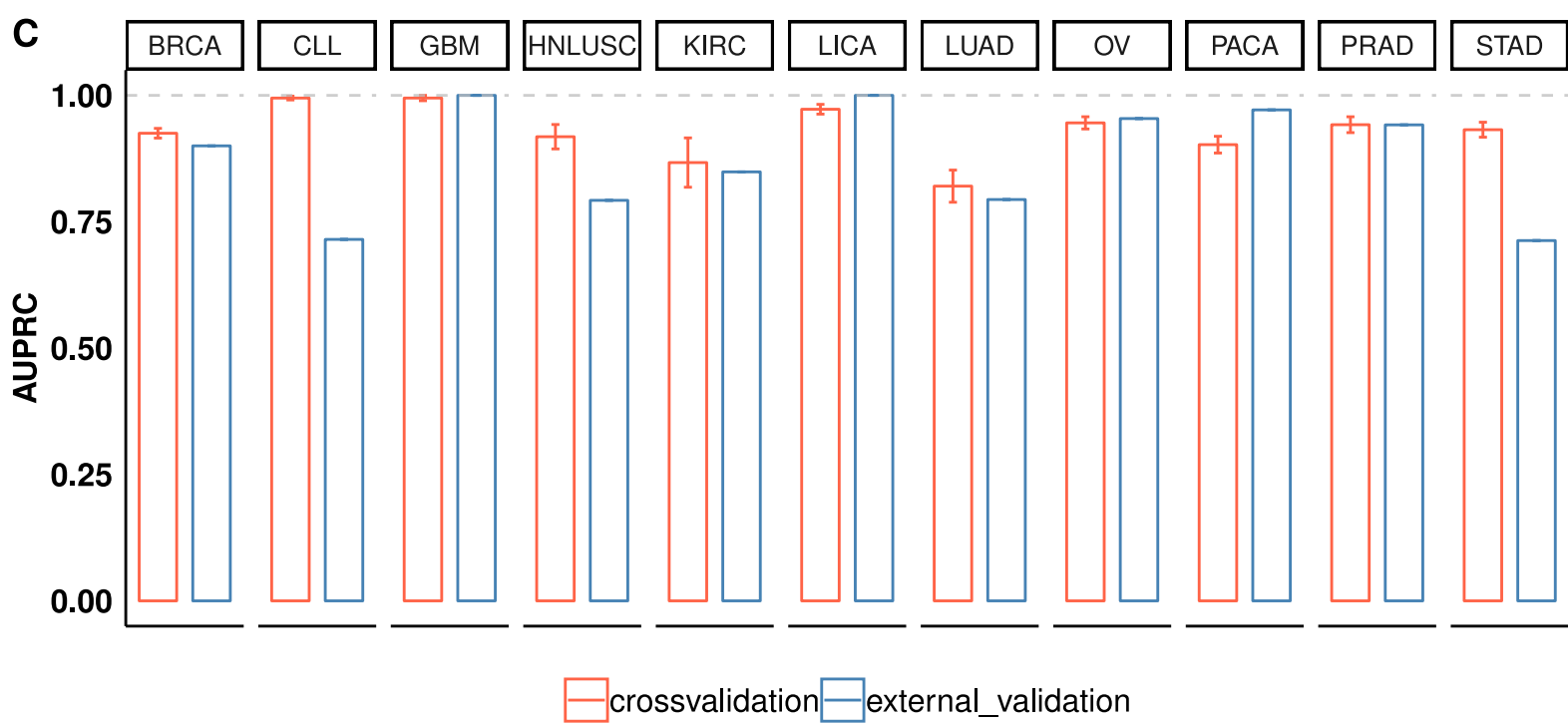

Supplement: S6 Fig — (A) Distribution of the total number of mutations per sample for each cancer type of the secondary training dataset (red) and the external validation dataset (blue). (B) For RMD features AUC mean of 5 independent runs obtained by training in the secondary training dataset and testing in the external validation dataset (blue) and by crossvalidation in the secondary training dataset (red) for each cancer type. Error bars represents the standard error of the mean of each cancer type. (C) For RMD features AUPRC mean of 5 independent runs obtained by training in the secondary training dataset and testing in the external validation dataset (blue) and by crossvalidation in the secondary training dataset (red) for each cancer type. Error bars represents the standard error of the mean of each cancer type. (PDF) [file pcbi.1006953.s006.pdf]

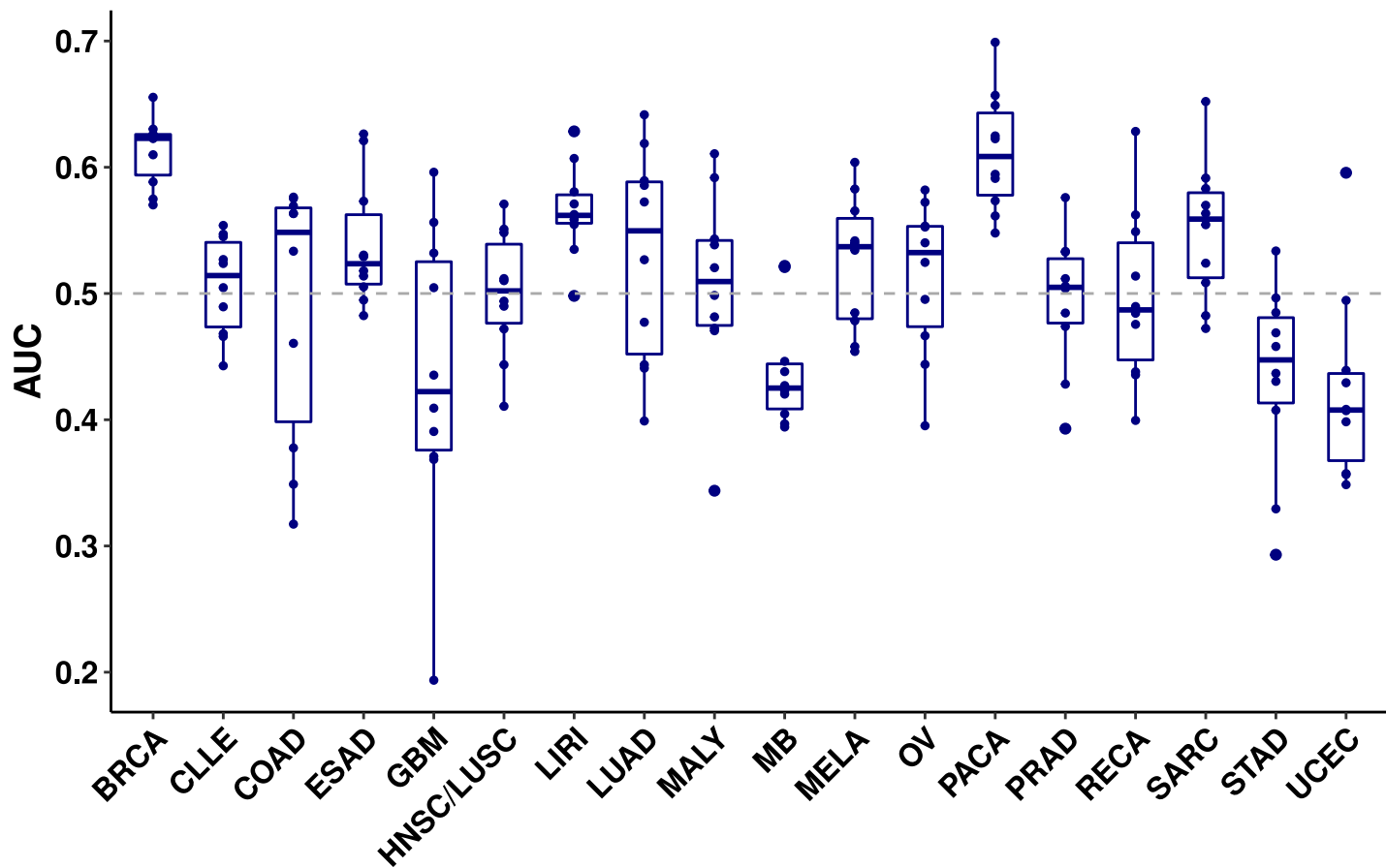

Supplement: S7 Fig — Area Under the ROC curve (AUC) for each cancer type calculated with RMD classifiers with the class labels (cancer type) randomized. Each dot corresponds to one independent run (10 runs in total). (PDF) [file pcbi.1006953.s007.pdf]

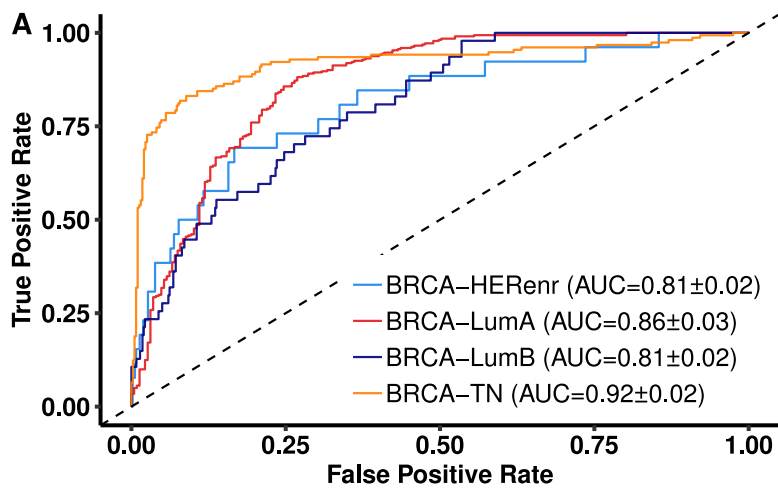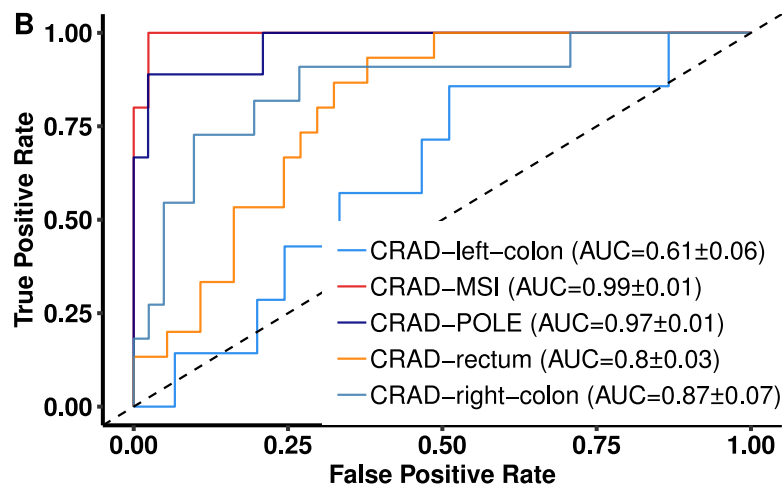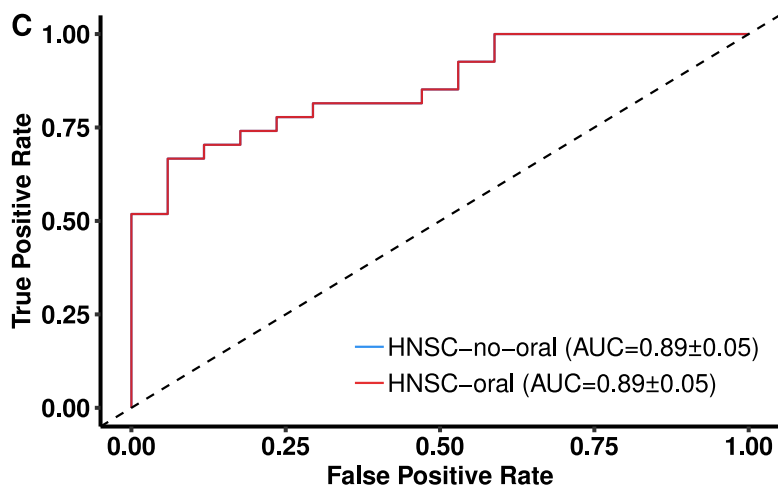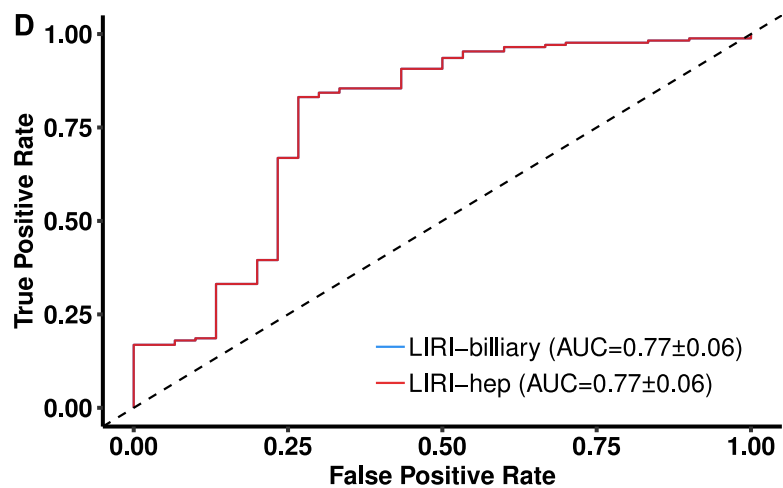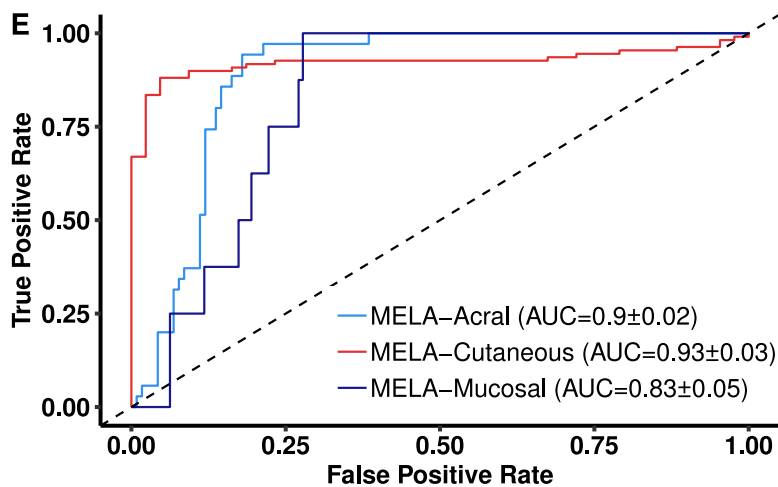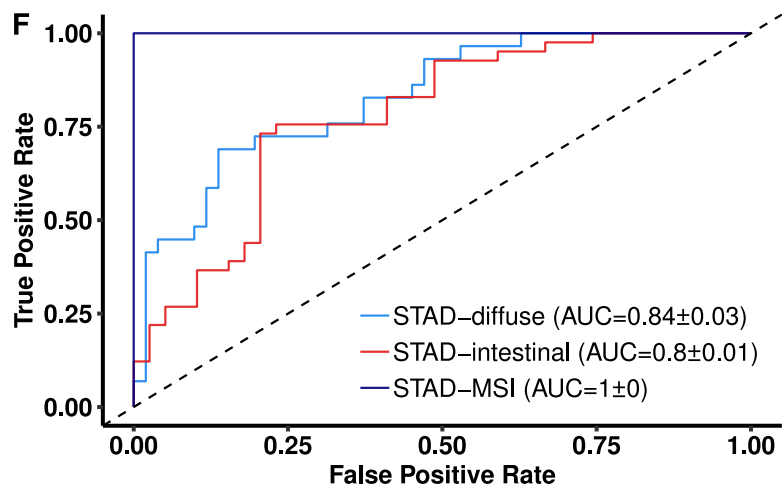

Supplement: S8 Fig — Receiver Operating Characteristic (ROC) curve for each subtype versus the rest of samples, in breast cancer (A), colorectal cancer (B), head and neck squamous cell adenocarcinoma (C), liver cancer (D), melanoma (E) and stomach cancer (F) datasets. Area Under the ROC curve (AUC) reported (mean and standard error of the mean across 5-fold cross validation rounds) in the legend of each panel. (PDF) [file pcbi.1006953.s008.pdf]

**A**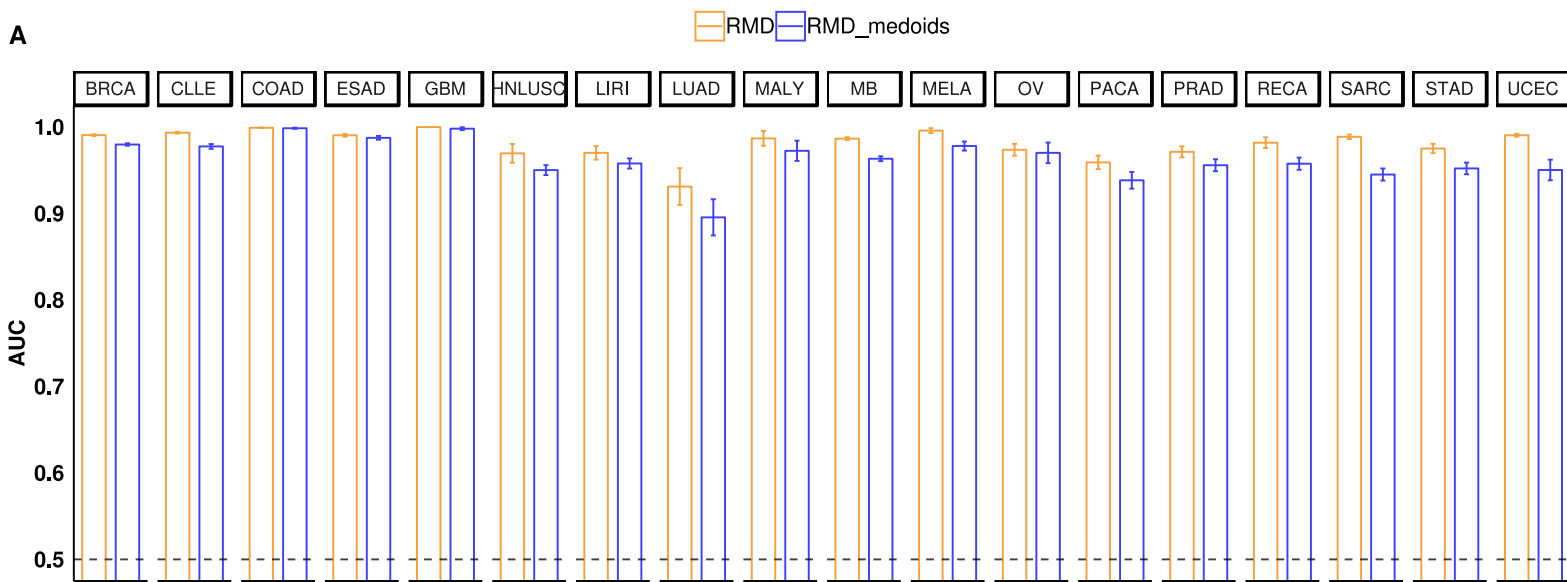**B**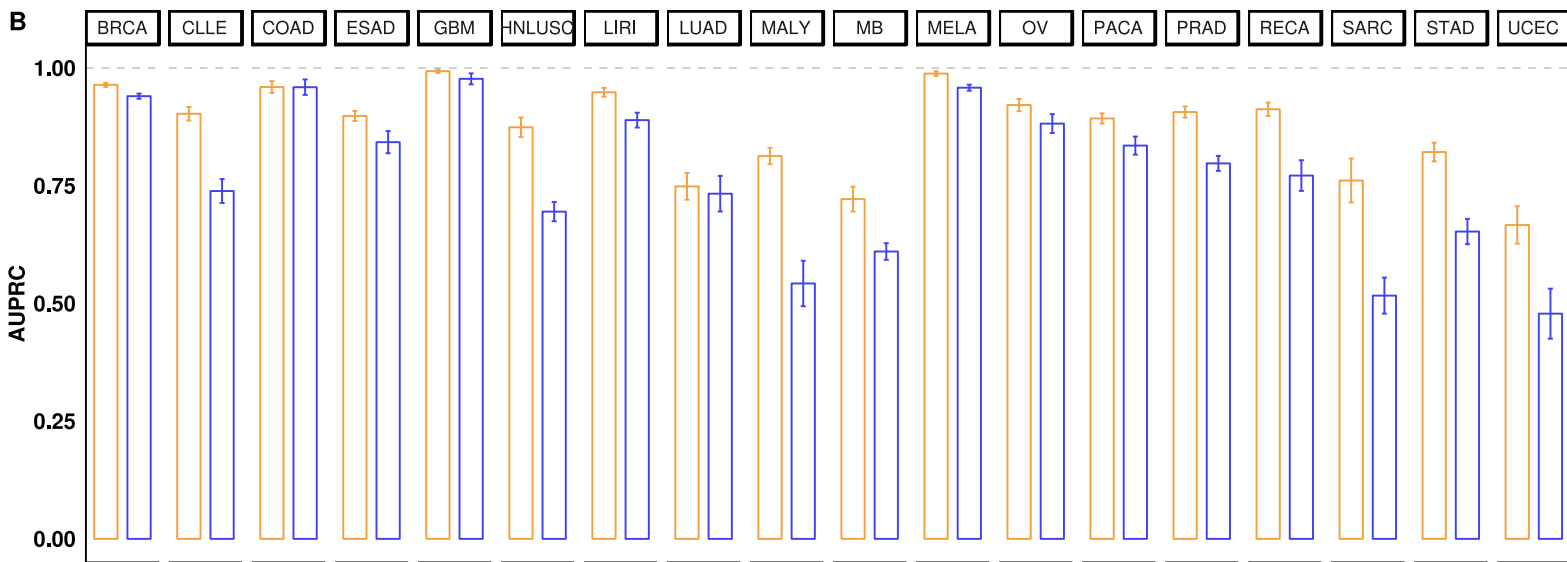

Supplement: S9 Fig — (A) Mean Area Under the ROC Curve (AUC) for each cancer type for the full set regional mutation density (RMD) features in yellow and the reduced set of 500 RMD features (by k-medoid clustering) in blue. (B) Mean Area Under the Precision Recall Curve (AUPRC) for each cancer type for the full set of RMD features in yellow and a reduced set of 500 RMD features (by k-medoid clustering) in blue. Error bars are the standard error of the mean. (PDF) [file pcbi.1006953.s009.pdf]

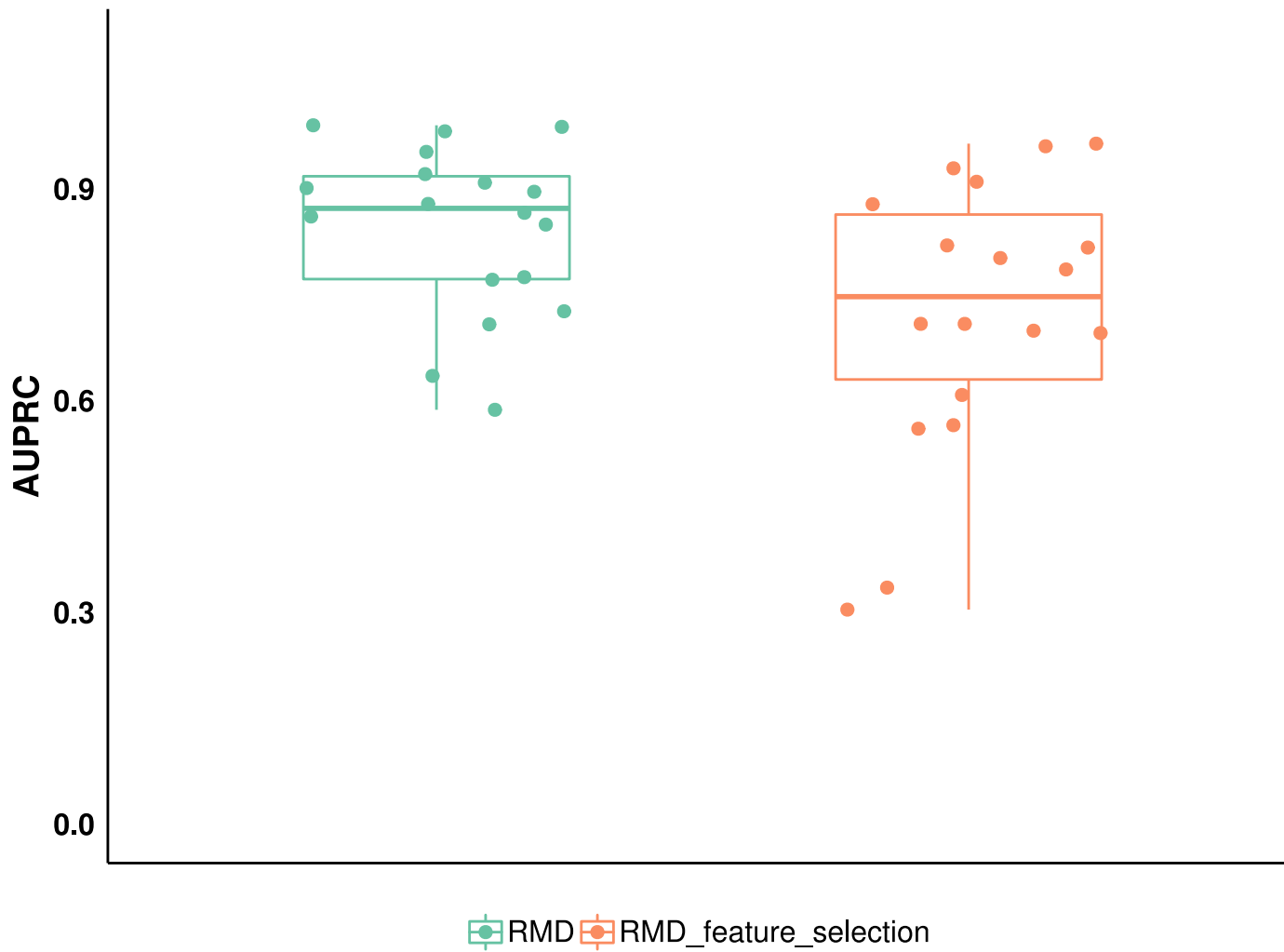

Supplement: S10 Fig — Area Under the Precision Recall Curve (AUPRC) for the main training dataset for each cancer type with all RMD features (in green); and applying feature selection to each training subset using Elastic Net and testing with those selected features within each fold of the crossvalidation (in orange). Each dot represents one cancer types (median AUPRC across the 3 folds of the crossvalidation, distinguishing that cancer type versus the rest of cancer types). (PDF) [file pcbi.1006953.s010.pdf]

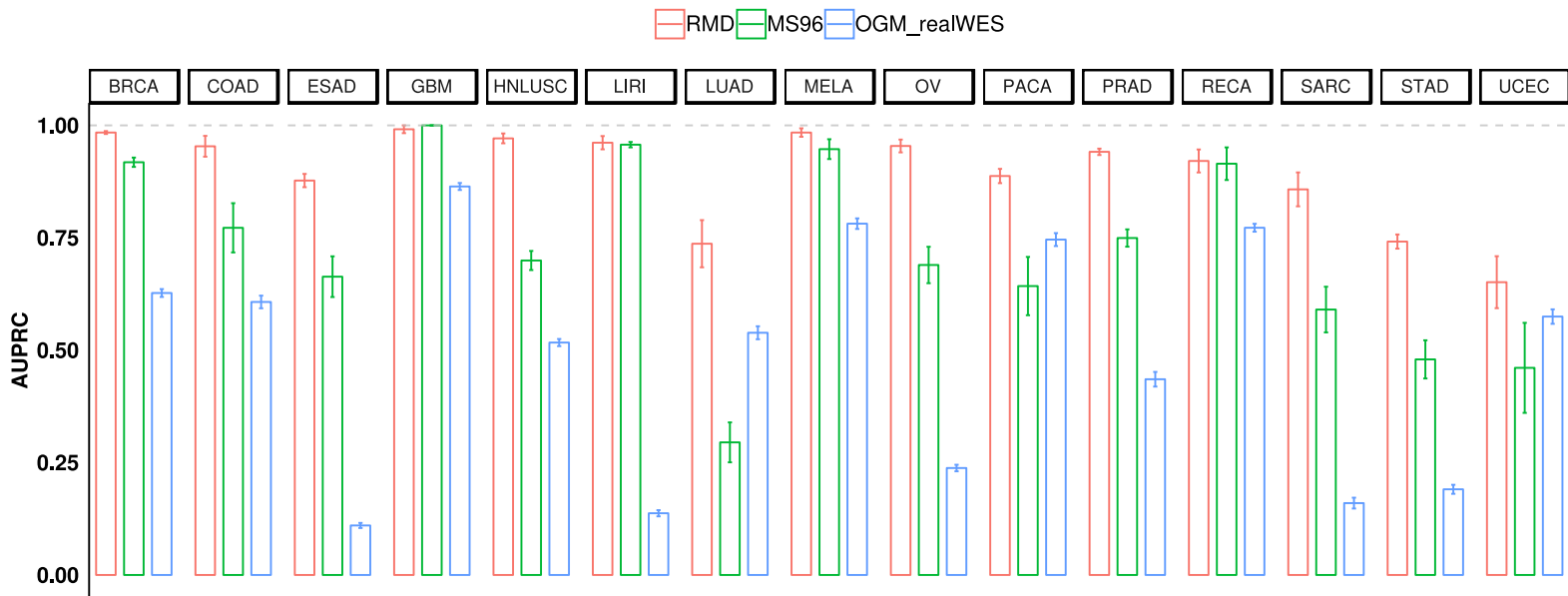

Supplement: S11 Fig — Mean Area Under the Precision Recall Curve (AUPRC) scores for each cancer type in the main training dataset, using regional mutation density (RMD) features in red, and 96 mutation spectra (MS96) features in green. The presence/absence of mutations in cancer genes (OGM features) was determined for a dataset of real WES with equivalent cancer types (shown in blue). (PDF) [file pcbi.1006953.s011.pdf]

**A**

OGM OGM\_cadd10 OGM\_cadd20 OGM\_sambar OGM\_weights

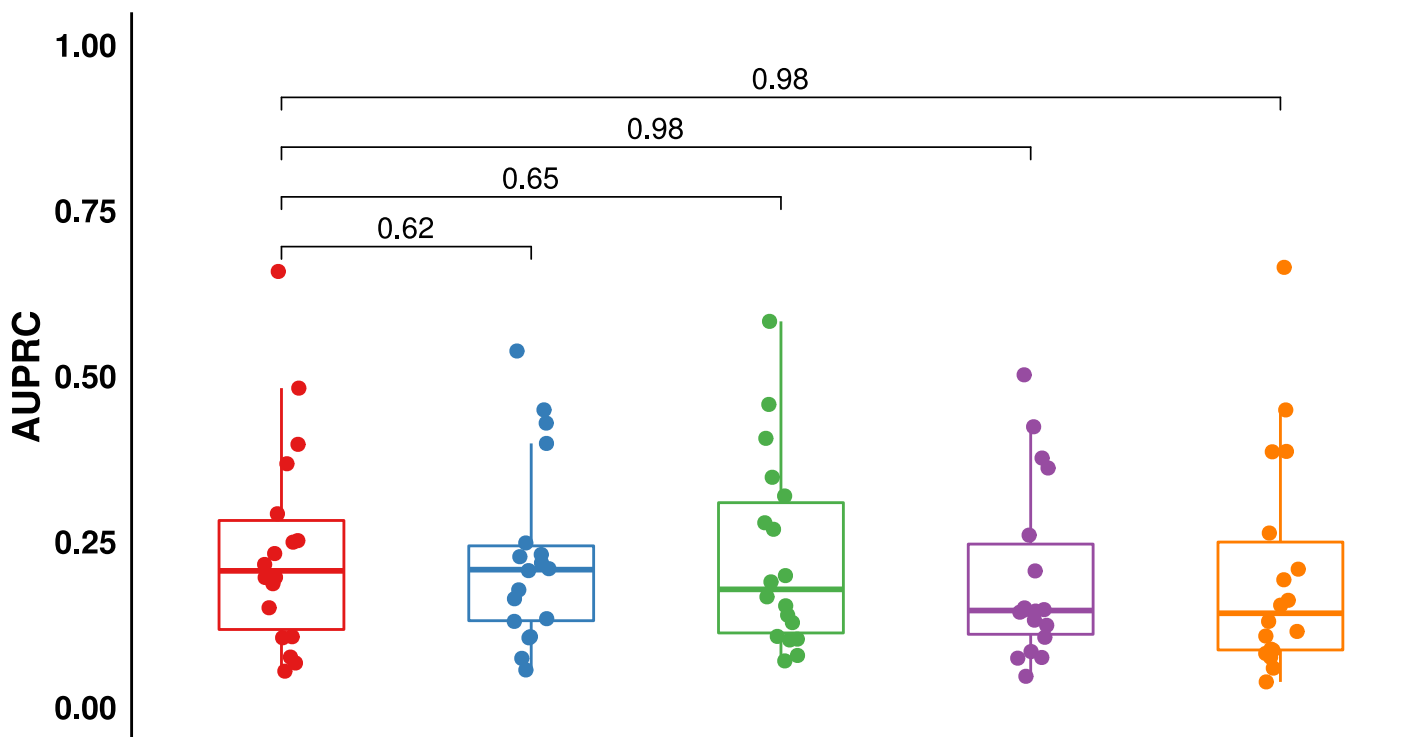**B**

OGM OGM\_Bailey OGM\_hotspots\_Bailey

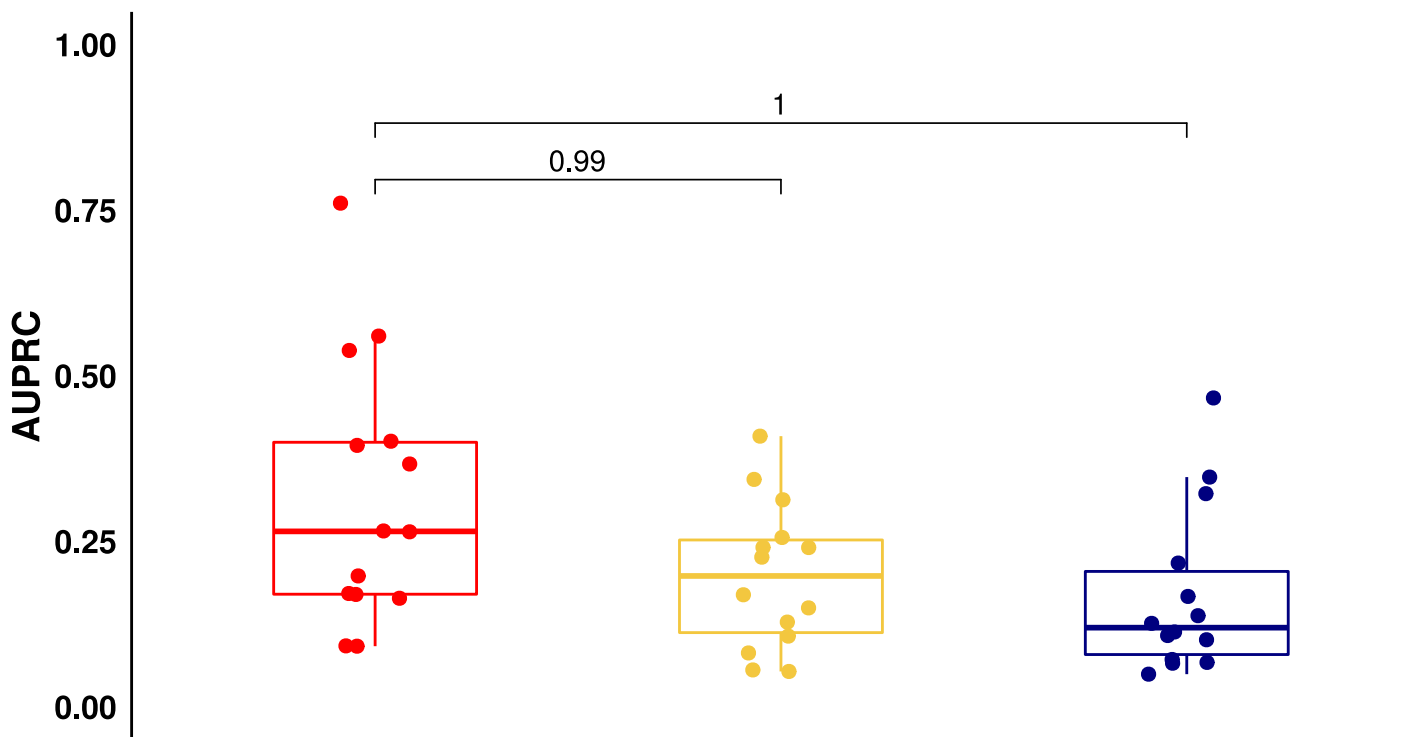

Supplement: S13 Fig — (A) Area Under the Precision Recall curve (AUPRC) for five sets of oncogenic mutation (OGM) features (see Methods) on WGS data. P-value reported for each set of features compares with the default OGM features as a baseline, using one-tailed Wilcoxon signed rank test (with alternative set to “less” in the R function wilcox.test). (B) Area Under the Precision Recall curve (AUPRC) for three different sets of OGM features (see Methods) in a subset of 560 patients (only considering genomes with at least one mutation from the Bailey et al. list). P-values for each set of features compare with the default OGM features as a baseline one, using a statistical test as in (A). (PDF) [file pcbi.1006953.s013.pdf]

OGM OGM\_cadd20 OGM\_weights OGM\_hotspots\_Bailey  
OGM\_cadd10 OGM\_sambar OGM\_Bailey

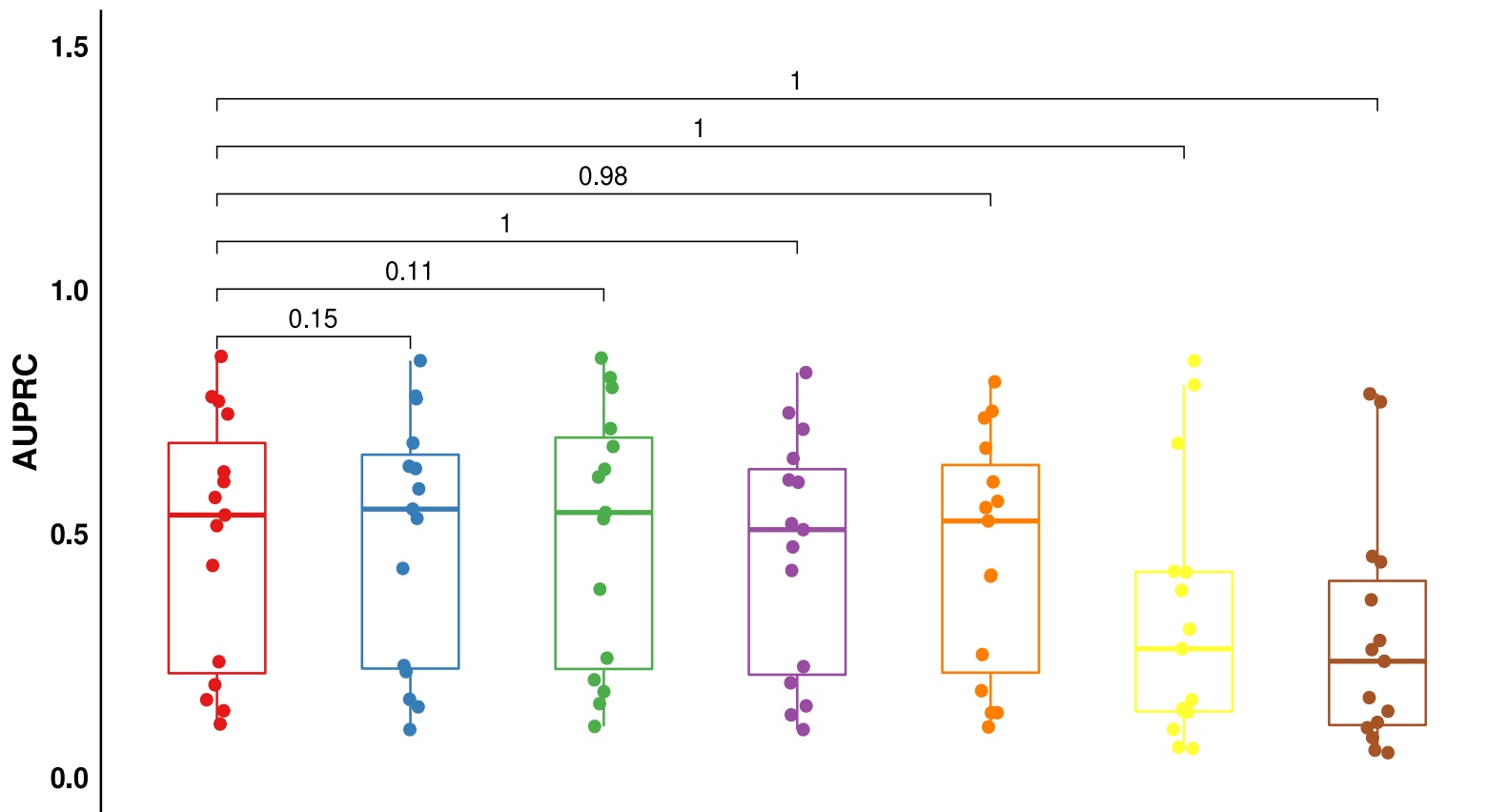

Supplement: S14 Fig — (A) Area Under the Precision Recall curve (AUPRC) for five sets of oncogenic mutation (OGM) features (see Methods) on WES data. P-value reported for each set of features compares against the baseline OGM features using a Wilcoxon signed rank test, one-tailed (alternative set to “less” in the R function wilcox.test). (PDF) [file pcbi.1006953.s014.pdf]

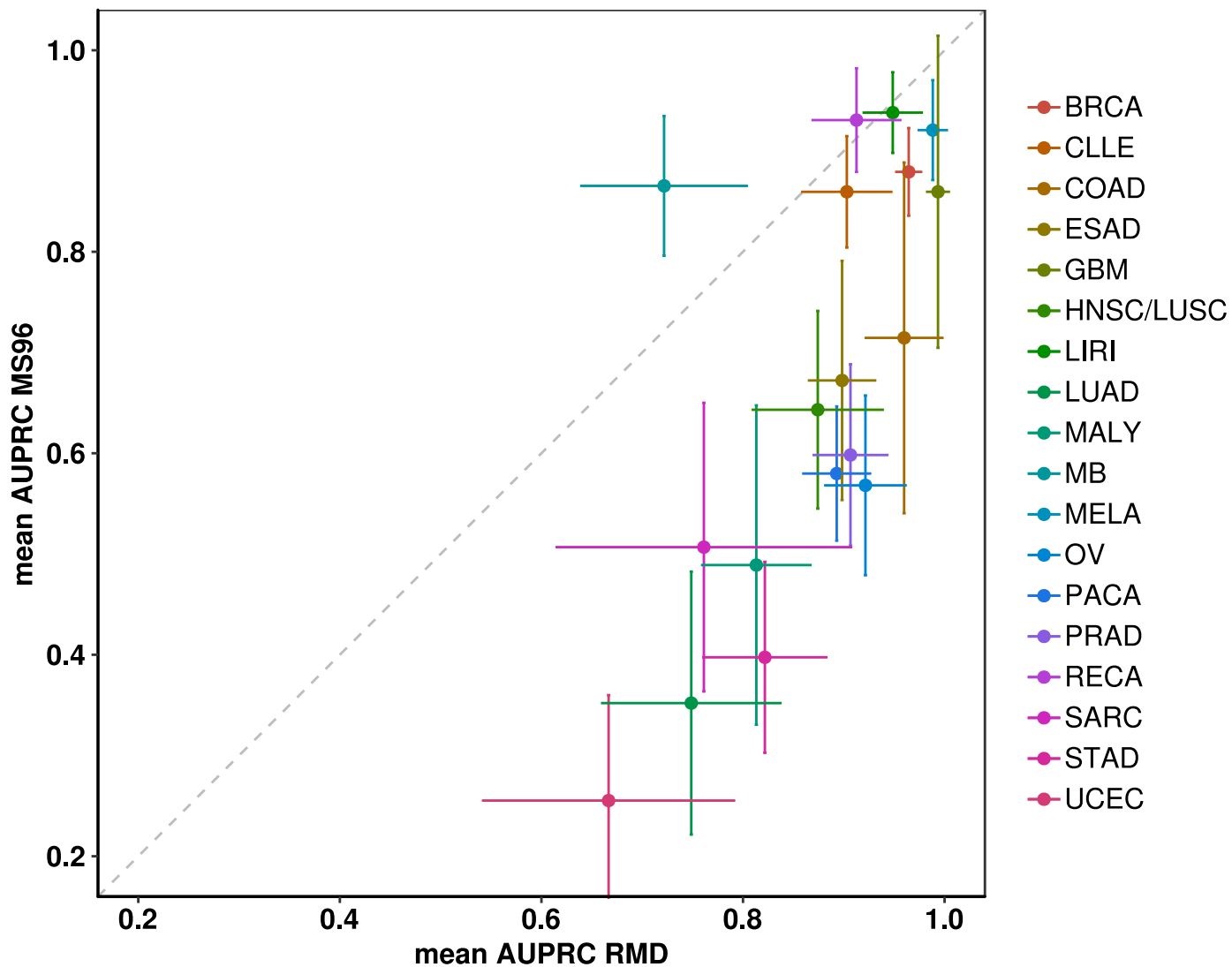

Supplement: S15 Fig — Mean Area Under the Precision Recall curve (AUPRC) score of the RMD features (x axis) versus MS96 features (y axis) of the main training dataset, in crossvalidation. Error bars are the standard deviation of AUPRC for RMD features (x axis) and MS96 features (y axis). (PDF) [file pcbi.1006953.s015.pdf]

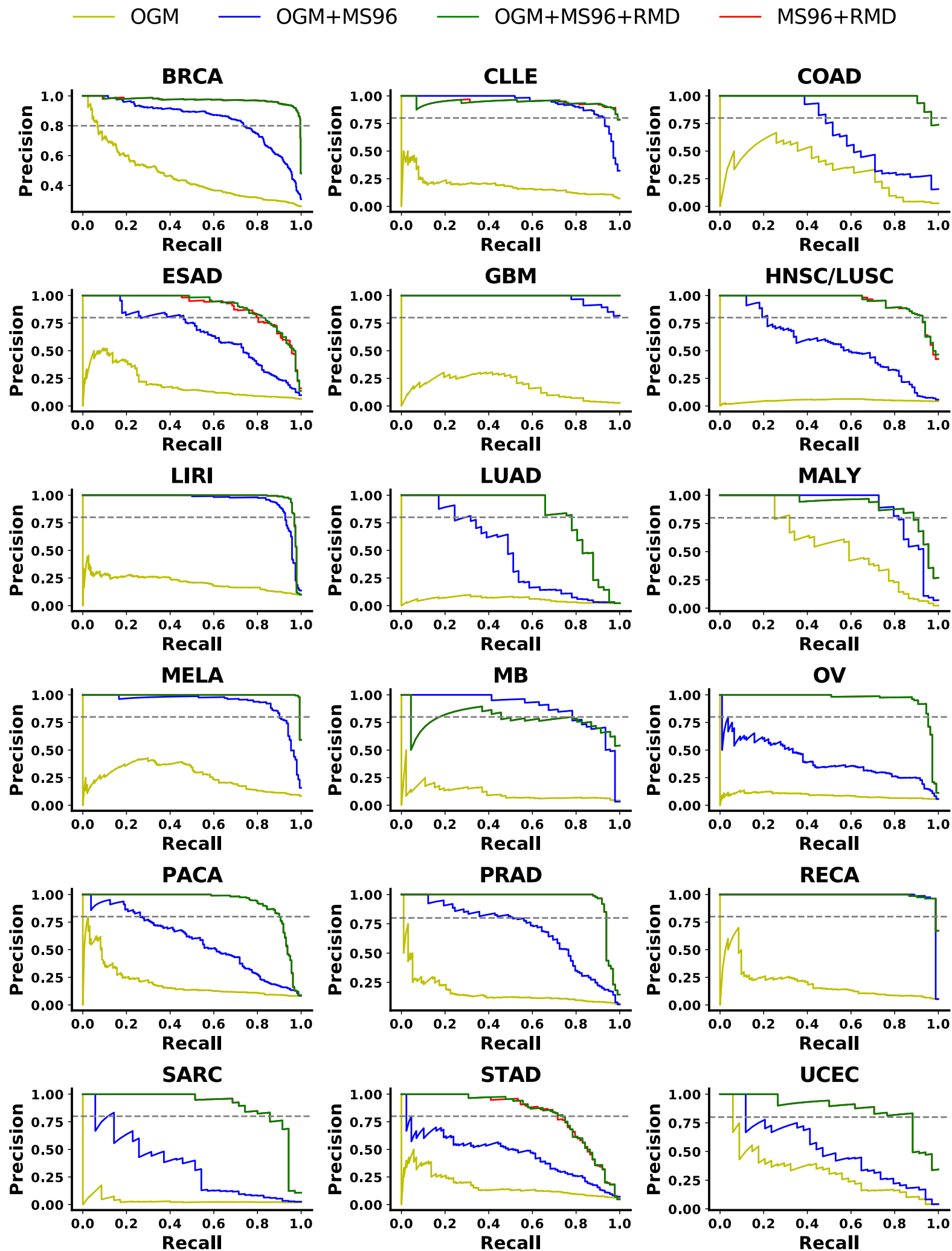

Supplement: S16 Fig — Precision-Recall Curve for each cancer type for the OGM (yellow), for the combination of OGM and MS96 (blue) and for the combination of the OGM, MS96 and the RMD (green), and MS96 and RMD without OGM (red) features for the main training dataset. In most cancer types the red curve overlaps the green curve perfectly and is thus hidden on the plots. Grey line indicates the threshold where precision = 0.8. (PDF) [file pcbi.1006953.s016.pdf]

— RMD — RMD+MS96

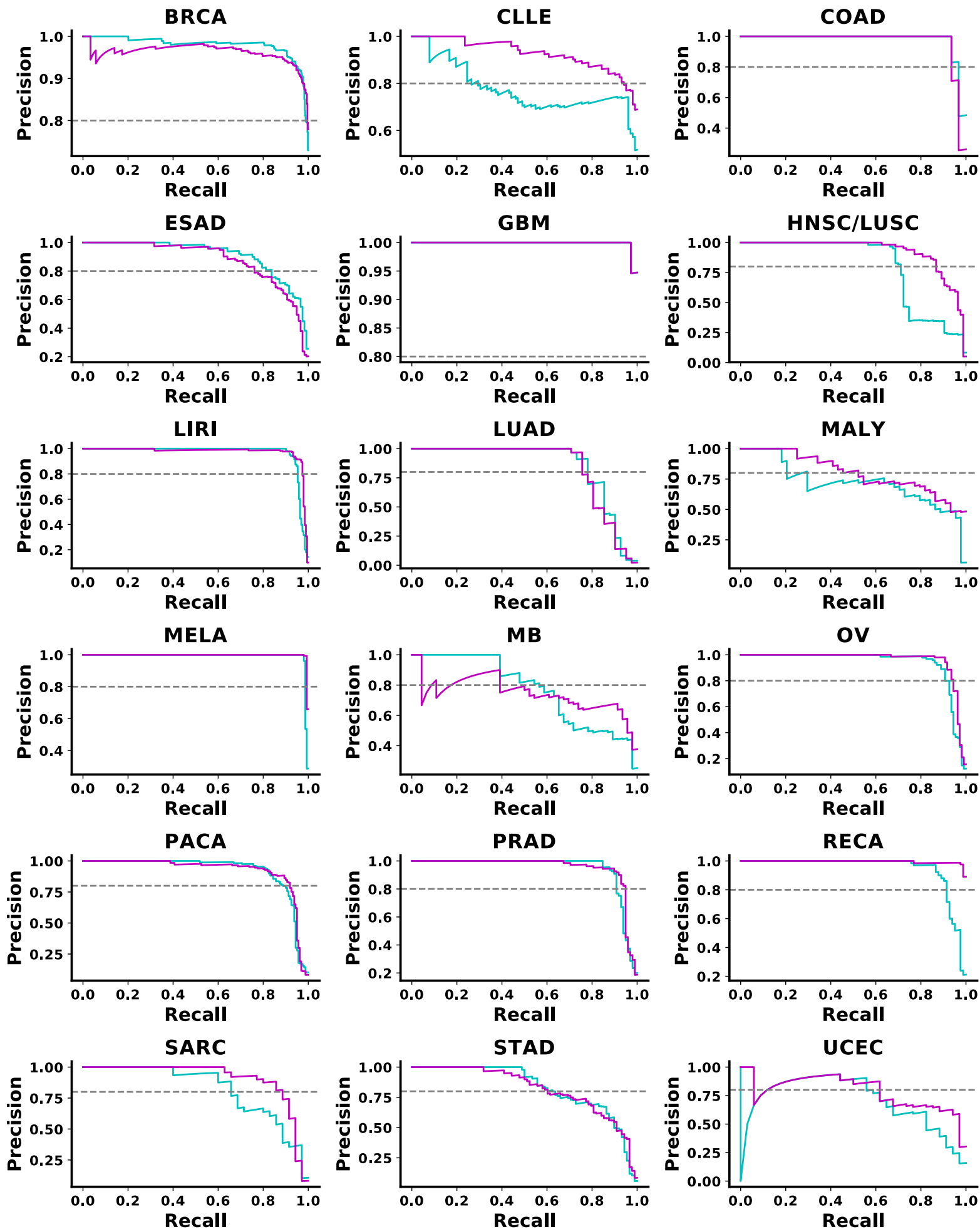

Supplement: S17 Fig — Precision-Recall Curve for each cancer type for the RMD (blue) and for the combination of RMD and MS96 (purple) for the main training dataset. Grey line indicates the threshold where precision is equal to 0.8 (PDF) [file pcbi.1006953.s017.pdf]

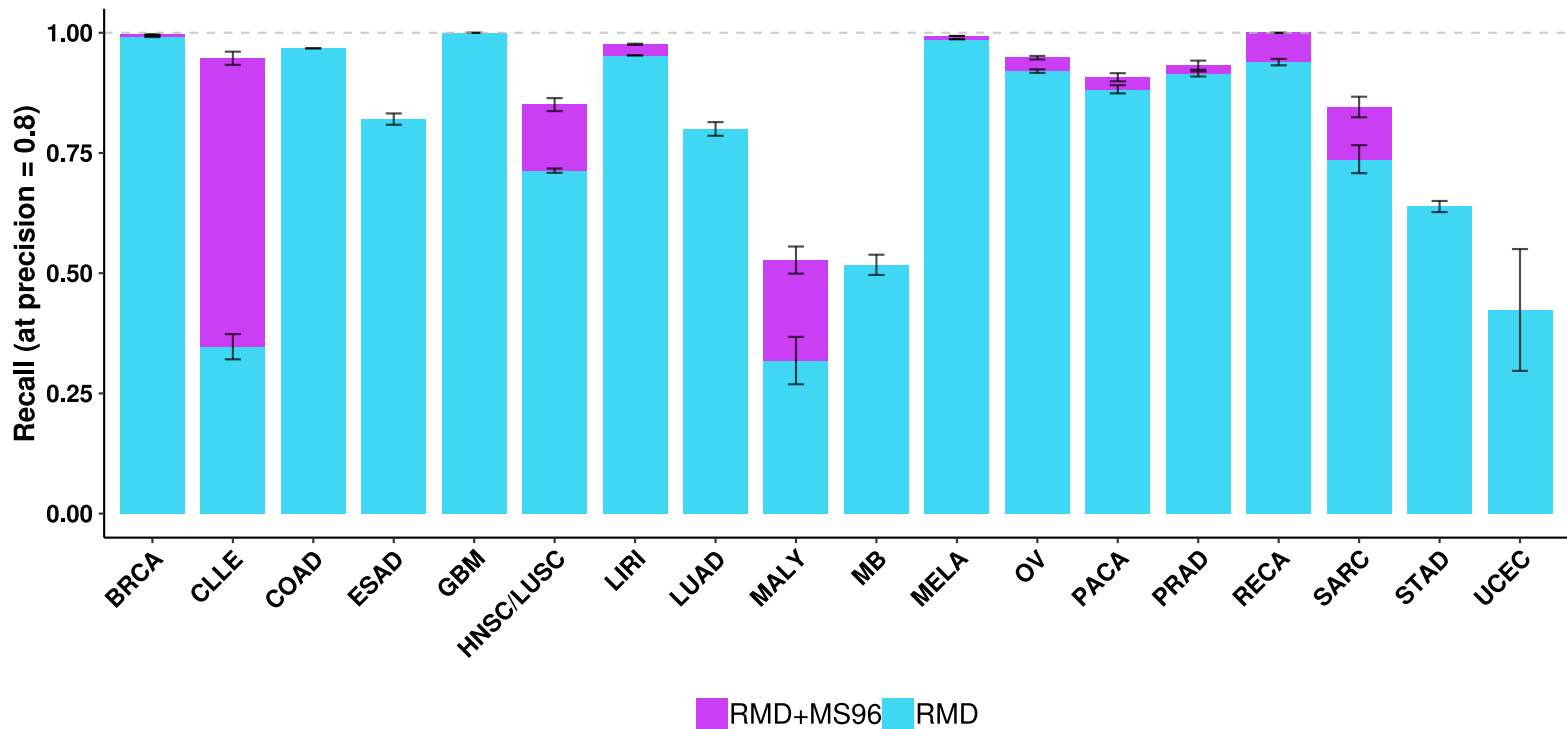

Supplement: S18 Fig — Recall at FDR = 20% for the classification models trained on RMD (blue) and RMD+MS96 features (purple); height of the stacked bars indicates the excess proportion of patients receiving correct predictions upon introducing the additional features to the classification model. Bars show the mean of five cross-validation runs, and error bars are standard deviations. (PDF) [file pcbi.1006953.s018.pdf]
