# Supplementary material for: Passenger mutations accurately classify human tumors
Source: PLoS Comput Biol. 2019 Apr 15;15(4):e1006953. doi: 10.1371/journal.pcbi.1006953 (PMC6483366; doi:10.1371/journal.pcbi.1006953)
Supplement: S12 Fig — (A) Mean Area Under the Precision Recall Curve (AUPRC) scores for each cancer type for the subtypes of 6 major cancer types, using different sets of features: regional mutation density (RMD) in red, 96 mutation spectra (MS96) in green and presence/absence of oncogenic mutations (OGM) in blue. (B) For the subtypes datasets of six cancer types % of samples that are: (i) correctly classified by both RMD and MS96 (yellow), (ii) misclassified by both methods (gray), (iii) correctly classified by the MS96 but not by the RMD (blue) and (iv) correctly classified by the RMD but not by the MS96 (red). (C) Venn diagram of samples correctly classified by MS96, RMD or OGM features and their intersections for the subtypes classification. (PDF) [file pcbi.1006953.s012.pdf]

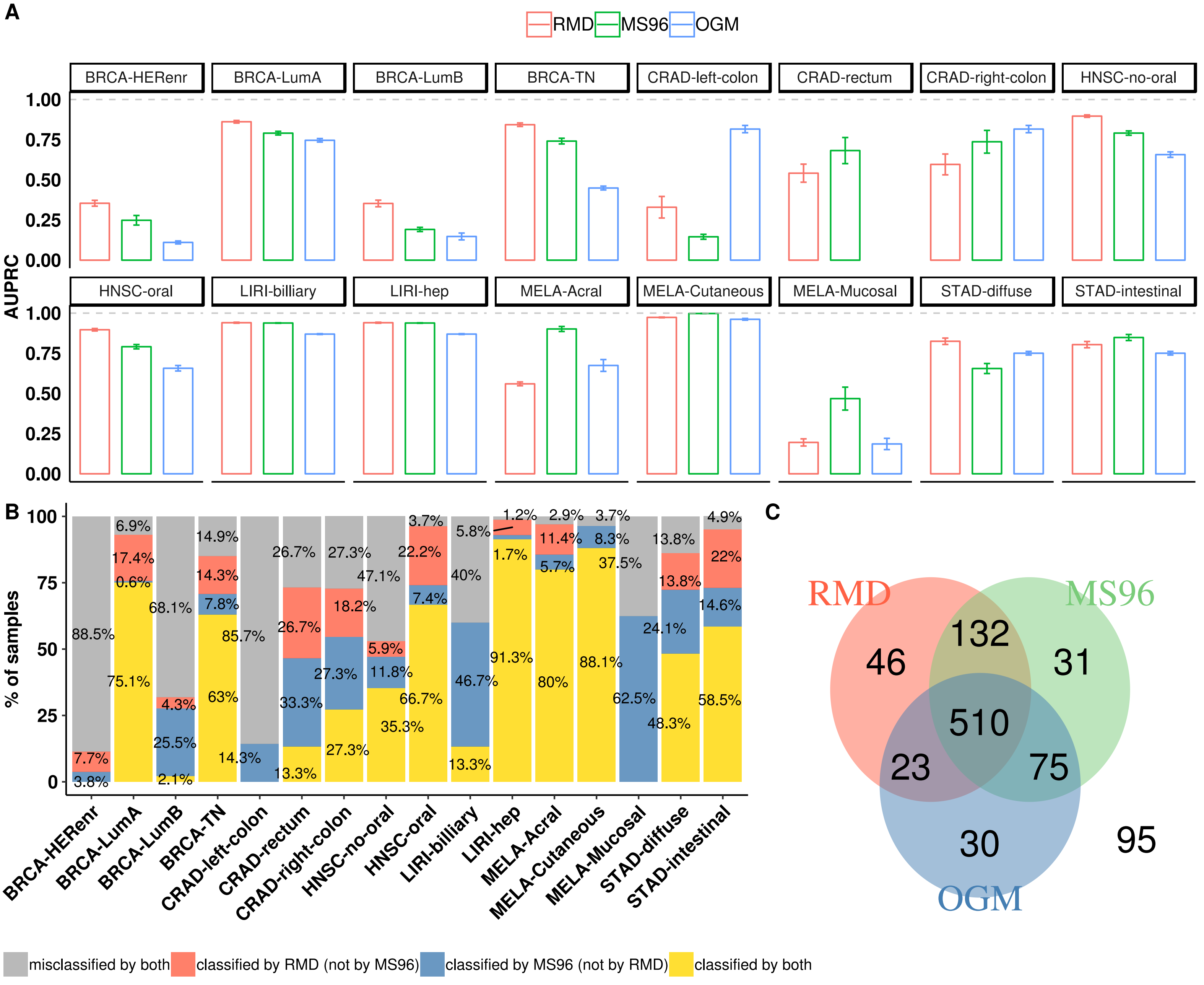

A
RMD
MS96
OGM
BRCA-HERenr
BRCA-LumA
BRCA-LumB
BRCA-TN
CRAD-left-colon
CRAD-rectum
CRAD-right-colon
HNSC-no-oral
HNSC-oral
LIRI-billiary
LIRI-hep
MELA-Acral
MELA-Cutaneous
MELA-Mucosal
STAD-diffuse
STAD-intestinal
AUPRC
1.00
0.75
0.50
0.25
0.00
B
% of samples
100
75
50
25
0
88.5%
7.7%
3.8%
6.9%
17.4%
0.6%
75.1%
68.1%
4.3%
25.5%
2.1%
14.9%
14.3%
7.8%
63%
14.3%
85.7%
26.7%
26.7%
33.3%
13.3%
27.3%
18.2%
27.3%
27.3%
47.1%
5.9%
11.8%
35.3%
3.7%
22.2%
7.4%
66.7%
5.8%
40%
46.7%
13.3%
1.2%
1.7%
91.3%
2.9%
11.4%
5.7%
80%
3.7%
8.3%
37.5%
88.1%
24.1%
62.5%
13.8%
13.8%
48.3%
4.9%
22%
14.6%
58.5%
misclassified by both
classified by RMD (not by MS96)
classified by MS96 (not by RMD)
classified by both
C
RMD
MS96
OGM
46
132
31
510
23
75
30
95
