# Supplementary material for: Passenger mutations accurately classify human tumors
Source: PLoS Comput Biol. 2019 Apr 15;15(4):e1006953. doi: 10.1371/journal.pcbi.1006953 (PMC6483366; doi:10.1371/journal.pcbi.1006953)
Supplement: S19 Fig — (A) For the main training dataset of simulated WES, fraction of samples that are: (1) correctly classified by both the passengers (RMD+MS96) and the drivers (OGM) (yellow), (2) misclassified by both methods (gray), (3) correctly classified by the passengers but not by the drivers (orange) and (4) correctly classified by the drivers but not by the passengers (green). (B) For RMD+MS96 features dataset, mean AUPRC of five classification runs obtained by training on the simulated WES dataset and testing on the real WES dataset as an external validation (blue) and by crossvalidation in the secondary training dataset (red) for each cancer type. Error bars represents the standard error of the mean of each cancer type. (C) As above, but for OGM features. Error bars represents the standard error of the mean of each cancer type. (PDF) [file pcbi.1006953.s019.pdf]

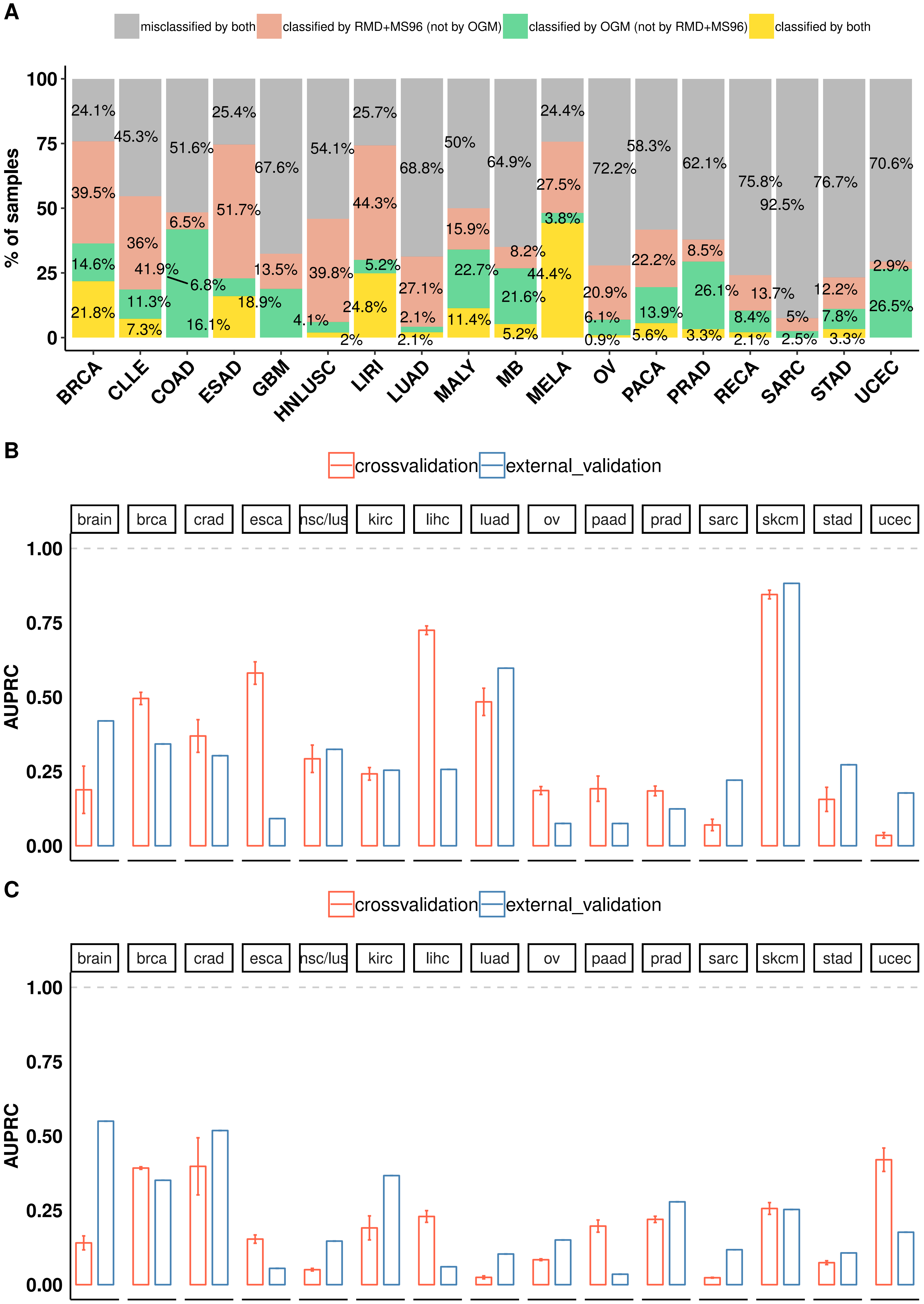
A
misclassified by both
classified by RMD+MS96 (not by OGM)
classified by OGM (not by RMD+MS96)
classified by both
% of samples
BRCA
CLLE
COAD
ESAD
GBM
HNLUSC
LIRI
LUAD
MALY
MB
MELA
OV
PACA
PRAD
RECA
SARC
STAD
UCEC
B
crossvalidation
external_validation
AUPRC
brain
brca
crad
esca
hsc/lus
kirc
lihc
luad
ov
paad
prad
sarc
skcm
stad
ucec
C
crossvalidation
external_validation
AUPRC
