# Supplementary material for: Passenger mutations accurately classify human tumors
Source: PLoS Comput Biol. 2019 Apr 15;15(4):e1006953. doi: 10.1371/journal.pcbi.1006953 (PMC6483366; doi:10.1371/journal.pcbi.1006953)
Supplement: S1 Text — (PDF) [file pcbi.1006953.s027.pdf]

Salvadores *et al*. (2019) PLOS Computational Biology.

Supplementary Methods

Collection and preparation of genomic data

We combined datasets from four different sources to maximize the coverage of cancer types and subtypes, encompassing 24 datasets with somatic mutations obtained by whole-genome sequencing (WGS), including: data from the ICGC Data portal [1], the WGS somatic single-nucleotide variants (SNVs) from Alexandrov *et al.* [2] and the somatic mutations of 100 WGS samples of stomach cancer from Wang *et al*. [3]. Additionally, data was obtained as aligned short reads (to the hg19/GRCh37 assembly) for the WGS of tumours and the matched normal tissue from the The Cancer Genome Atlas (TCGA) repository, formerly at CGHub (now decommissioned), and somatic mutations were called as described previously [4]. Finally, the files containing somatic mutations for whole-exome sequences (WXS) were downloaded from the Genomic Data Commons (TCGA). To minimize errors due to misalignment of short reads, we masked out all regions in the genome defined in the 'CRG Alignability 36' track [5] as having alignability <1.0, for all datasets.

For the WGS analysis, we created a main training dataset including all available cancer types with more than 20 tumor samples (S1 Table). LUSC and HNSC were merged into a single category called HNSC/LUSC [6]. For validation, we examined all cancer types from the main training dataset that had an equivalent dataset from a different data source or sequencing center, which therefore constitute the external validation dataset (S2 Table). Additionally, we separated six cancer types into subtypes based on molecular subtyping, their microsatellite instability (MSI) status and presence of cancer-associated viruses HPV for HNSC or EBV for STAD (S3 Table). For the analysis of robustness towards noise, we simulated genomes with false negative mutation calls (dropout) by randomly sampling 100, 50, 25, 5, 2, 1 and 0.1% of the mutations in each tumor.

Additionally, we simulated WXS data from the WGS datasets by retaining only mutations inside the regions that reflect the actual WXS sequencing coverage for the three TCGA sequencing centers: BI, BCM and WUGSC, requiring at least 8 reads in at least 90% samples from each sequencing center, as previously [7]. Of note, we used the intersection of these three regions to reduce biases when comparing cancer types from different sequencing centers. This filtering is more stringent (~1.8% mutations retained) than using only the WXS regions from one sequencing center (~2.2% mutations retained). Regarding validation of the WXS analyses, we used the same main training dataset for generating simulated WXS data to train the classification model, which were then validated by comparing with tissue-matched real WXS from TCGA, which thus constituted an external validation dataset (Supplementary Table 4).

Obtaining Copy Number Alteration (CNA) data

We downloaded CNAs data (SNP6) from FireBrowse [8]. For each CNA segment we use a threshold of segment_mean>0.1 and <-0.1 to classify the segment as +1 or -1 respectively (the rest were set to 0). For each sample, the CNAs matrix was calculated dividing each chromosome into 1 Mb windows, counting the number of CNA events per window and labelling them as +1 if amplification counts was higher or -1 if deletion counts was higher in the window. Afterwards, we divided the CNA matrix into CNA drivers and CNA passengers matrices, where drivers affect the 1 Mb windows containing the 64 known dosage-sensitive cancer genes from the Cancer Gene Census (68 features). The passengers are the remaining CNA that do not overlap the windows containing those genes nor are in their immediate vicinity (excluding 1 Mb either side) (2715 features).

Supplementary Results

Copy number alteration-based tumor type classifiers

Our analysis up to this point adressed the distribution of mutations encompassing single-nucleotide variants (SNVs) and small indels. This is because previous work suggested that SNVs and indels might convey tissue-specific signal in form of trinucleotide spectra [2] and in form of domain-scale mutation rates [4,9]. Another very common type of genetic change in tumors are somatic copy-number alterations (CNA), constituting amplifications or deletions affecting cancer driver genes which are dosage-sensitive [10] and additionally many other alterations which have a lower or no selective advantage. It is indeed known that some CNA recur more often in certain cancer types, providing a rationale for previous use of CNA features for tumor type classification [11,12]. We briefly investigated their predictive power, comparing it to the mutational features derived from driver mutations and from global patterns of passenger mutations.

We tentatively divided the CNA into drivers and passengers, where drivers affect the 64 known dosage-sensitive cancer genes from the Cancer Gene Census. The passengers are the remaining CNA that do not overlap those genes nor are in their immediate vicinity (1 Mb either side), and are represented as copy-numbers in megabase-sized windows across the genome, by analogy with RMD features. An important consideration is that it is very difficult to resolve driver from passenger CNA events because some CNA may span very large swaths of a chromosome, affecting many genes – drivers and passengers alike – and therefore the signal we observe will necessarily be mixed. Furthermore it has been argued that the copy number changes that affect many genes that are not conventional drivers may still have selective advantages to the tumor [13]. Another consideration is that the CNA estimates we analyze are obtained from SNP arrays (from the TCGA project) while in realistic applications, such as liquid biopsies, the CNAs would often be estimated from DNA sequencing [14]. This may not yield similar quality CNA estimates, particulary in case of exome or panel sequencing.

By analogy with examining mutational features, we find that driver CNA are less predictive of cancer type than the putative passenger CNA profiles, with AUPRC 0.46 and 0.75 (median across 15 cancer types) for drivers versus passengers (Fig S15). On a matched set of whole genome sequences from 15 cancer types, we observed median AUPRC 0.22 for SNV/indel drivers (OGM) versus 0.92 for SNV/indel passengers (RMD+MS96). Overall, two trends emerge. First, both for CNA and for SNV/indels, the genome-wide pattern emanating from non-selected alterations provides a better tumor classifier than the known oncogenic CNAs, SNVs or indels. Second, that CNA in fact have a high potential for classifying cancer type, at least when measured on high-purity tumor samples using SNP arrays. Further analyses will elucidate determine how the CNA classifiers fare on lower quality data; simulation studies may prove useful in this task [11,12].

Supplementary Text References

1. The International Cancer Genome Consortium. International network of cancer genome projects. Nature. Nature Publishing Group; 2010;464: 993–998. doi:10.1038/nature08987

2. Alexandrov LB, Nik-Zainal S, Wedge DC, Aparicio SAJR, Behjati S, Biankin A V., et al. Signatures of mutational processes in human cancer. Nature. Nature Publishing Group; 2013;500: 415–421. doi:10.1038/nature12477

3. Wang K, Yuen ST, Xu J, Lee SP, Yan HHN, Shi ST, et al. Whole-genome sequencing and comprehensive molecular profiling identify new driver mutations in gastric cancer. Nat Genet. Nature Publishing Group; 2014;46: 573–582. doi:10.1038/ng.2983

4. Supek F, Lehner B. Differential DNA mismatch repair underlies mutation rate variation across the human genome. Nature. Nature Publishing Group; 2015;521: 81–84. doi:10.1038/nature14173

5. Derrien T, Estellé J, Marco Sola S, Knowles DG, Raineri E, Guigó R, et al. Fast Computation and Applications of Genome Mappability. Ouzounis CA, editor. PLoS One. Public Library of Science; 2012;7: e30377. doi:10.1371/journal.pone.0030377

6. Hoadley KA, Yau C, Wolf DM, Cherniack AD, Tamborero D, Ng S, et al. Multiplatform analysis of 12 cancer types reveals molecular classification within and across tissues of origin. Cell. NIH Public Access; 2014;158: 929–944. doi:10.1016/j.cell.2014.06.049

7. Park S, Supek F, Lehner B. Systematic discovery of germline cancer predisposition genes through the identification of somatic second hits. Nat

Commun. Nature Publishing Group; 2018;9: 2601. doi:10.1038/s41467-018-04900-7

8. Broad Institute of MIT and Harvard. Broad Institute TCGA Genome Data Analysis Center. In: Firehose stddata__2016_01_28 run [Internet]. 2016 [cited 24 Aug 2018]. doi:doi:10.7908/C11G0KM9

9. Polak P, Karlić R, Koren A, Thurman R, Sandstrom R, Lawrence MS, et al. Cell-of-origin chromatin organization shapes the mutational landscape of cancer. Nature. Nature Publishing Group; 2015;518: 360–364. doi:10.1038/nature14221

10. Zack TI, Schumacher SE, Carter SL, Cherniack AD, Saksena G, Tabak B, et al. Pan-cancer patterns of somatic copy number alteration. Nat Genet. Nature Publishing Group; 2013;45: 1134–1140. doi:10.1038/ng.2760

11. Marquard AM, Birkbak NJ, Thomas CE, Favero F, Krzystanek M, Lefebvre C, et al. TumorTracer: a method to identify the tissue of origin from the somatic mutations of a tumor specimen. BMC Med Genomics. BioMed Central; 2015;8: 58. doi:10.1186/s12920-015-0130-0

12. Molparia B, Nichani E, Torkamani A. Assessment of circulating copy number variant detection for cancer screening. Galli A, editor. PLoS One. 2017;12: e0180647. doi:10.1371/journal.pone.0180647

13. Davoli T, Xu AW, Mengwasser KE, Sack LM, Yoon JC, Park PJ, et al. Cumulative haploinsufficiency and triplosensitivity drive aneuploidy patterns and shape the cancer genome. Cell. Elsevier; 2013;155: 948–62. doi:10.1016/j.cell.2013.10.011

14. Adalsteinsson VA, Ha G, Freeman SS, Choudhury AD, Stover DG, Parsons HA, et al. Scalable whole-exome sequencing of cell-free DNA reveals high concordance with metastatic tumors. Nat Commun. Nature Publishing Group; 2017;8: 1324. doi:10.1038/s41467-017-00965-y
